# The Crosstalk Between CRL5 and APC/C E3 Ligases Regulates Metastasis and Chemosensitivity of Cancer Cells

**DOI:** 10.1002/advs.202512652

**Published:** 2025-10-29

**Authors:** Danrui Cui, Ruirui Qu, Tianqi Li, Linchen Wang, Xiaoyu Chen, Shengpeng Shao, Xue Liang, Jun Xu, Yi Sun, Xiufang Xiong, Yongchao Zhao

**Affiliations:** ^1^ Department of Hepatobiliary and Pancreatic Surgery the First Affiliated Hospital Zhejiang University School of Medicine Hangzhou 310003 China; ^2^ Zhejiang Provincial Key Laboratory of Pancreatic Disease the First Affiliated Hospital Zhejiang University School of Medicine Hangzhou 310003 China; ^3^ Institute of Translational Medicine Zhejiang University School of Medicine Hangzhou 310029 China; ^4^ Zhejiang Key Laboratory of Frontier Medical Research on Cancer Metabolism Hangzhou 310029 China; ^5^ Department of Medical Oncology the First Affiliated Hospital Zhejiang University School of Medicine Hangzhou 310003 China; ^6^ Cancer Institute of the Second Affiliated Hospital Zhejiang University School of Medicine Hangzhou 310029 China; ^7^ Cancer Center Zhejiang University Hangzhou 310029 China

**Keywords:** APC11, chemosensitivity, CUL5, metastasis, neddylation

## Abstract

Cullin‐RING ligases (CRLs) and the anaphase‐promoting complex/cyclosome (APC/C) are two major multi‐subunit ubiquitin ligases essential for protein homeostasis. The underlying mechanism and biological consequence of their crosstalk remain elusive. Here, tandem affinity purification followed by LC‐MS/MS is employed, and identified APC11—the RING subunit of APC/C—as a bona fide binding partner of CUL5, the scaffold of CRL5. On one hand, APC11 interacts with CUL5 and inhibits its neddylation. Consequently, APC11 knockdown enhances CUL5 neddylation by promoting its interaction with the neddylation E2 UBE2F. This leads to the degradation of SOCS3, a substrate receptor of CRL5, and the subsequent accumulation of its substrate, integrin β1, ultimately promoting cancer metastasis. On the other hand, CUL5‐APC11 binding stabilizes APC11 by facilitating its atypical K27/K29/K33‐linked polyubiquitylation at Lys83, a process catalyzed by ITCH E3 ligase. CUL5 loss delays mitotic exit, induces aneuploidy, and sensitizes cancer cells to the microtubule‐targeting drug paclitaxel by destabilizing APC11. Collectively, the study revealed a new crosstalk between CUL5 and APC11 of two major E3 ligases, and targeting this crosstalk can provide a new strategy for blocking metastasis and triggering chemosensitization.

## Graphical abstract

This study uncovers a previously unknown crosstalk between two major ubiquitin ligases, CRL5 and APC/C. The authors identify APC11 as a binding partner of CUL5, showing that their interaction regulates neddylation, substrate stability, and mitotic progression. Disruption of this axis drives metastasis and alters paclitaxel sensitivity, suggesting therapeutic opportunities in cancer treatment.

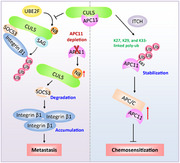

## 1 Introduction

Ubiquitylation is a complex tripartite enzymatic process involving the ubiquitin activating enzyme (E1), the ubiquitin conjugating enzyme (E2), and the ubiquitin ligase (E3), which collectively catalyzes the attachment of the ubiquitin to a substrate protein. In addition to canonical lysine ubiquitylation, non‐canonical ubiquitylation also occurs on cysteine, serine, and threonine residues, as well as on the free amino group at the protein N‐terminus.^[^ ^1^, ^2^ ^]^ While most ubiquitin modifications target substrates for degradation via the ubiquitin‐proteasome system (UPS), they also exert non‐proteolytic functions in diverse cellular processes, such as signal transduction, cell division, and DNA damage response.^[^ ^3^, ^4^ ^]^

Multiple ubiquitin molecules form polyubiquitin chains by linking through one of seven lysine residues (K6, K11, K27, K29, K33, K48, and K63) or the N‐terminal methionine residue (M1), making ubiquitylation a highly versatile and prevalent post‐translational modification in cells. While the roles of K11‐ and K48‐linked chains in proteasomal degradation, as well as those of linear (M1) and K63‐linked chains in signal transduction, are well‐established, the function of “atypical” K6, K27, K29, and K33‐linkages remains largely unclear.^[^ ^5^, ^6^, ^7^, ^8^ ^]^ In addition, certain proteins undergo monoubiquitylation at a single residue or at multiple sites, thereby regulating protein–protein interactions, subcellular localization, and activity.^[^ ^9^ ^]^

In humans, the ubiquitin system comprises ≈1500 components, about half of them (>600) are E3 ligases (E3s) that specifically and selectively recognize their substrates.^[^ ^10^, ^11^ ^]^ Among all E3s, cullin‐RING ligase (CRL) and the anaphase promoting complex/cyclosome (APC/C) are the only two multi‐subunit ubiquitin ligase families within the RING finger‐containing E3 class.^[^ ^12^, ^13^ ^]^ The regulation of CRLs and APC/C E3 ligases is crucial, as cells need to maintain the correct balance of subunits within multi‐protein complexes to ensure proper protein quality control.^[^ ^14^ ^]^

CRLs, the largest family of E3 ubiquitin ligases, consist of cullin scaffolds, RING finger proteins, adaptor proteins, and substrate receptors. These complexes catalyze the ubiquitylation of ≈20% cellular proteins that are degraded through the UPS.^[^ ^15^ ^]^ In the CRL5 complex, cullin 5 (CUL5) serves as the molecular scaffold, with elongin B/C adaptor proteins and a SOCS box protein binding to its N‐terminus, and RBX2 (also known as SAG or ROC2) interacts with its C‐terminus. SAG, a RING protein, primarily binds to CUL5 rather than other cullins, recruiting ubiquitin‐loaded E2 (such as UBE2C and UBE2S) to facilitate the transfer of ubiquitin from E2 to the substrate recognized by SOCS.^[^ ^16^ ^]^ The activation of CRLs is predominantly regulated by neddylation, a ubiquitin‐like modification in which the NEDD8 molecule is covalently conjugated to a conserved lysine residue at the C‐terminus of cullins.^[^ ^17^ ^]^ In the case of CUL5, SAG also functions as a neddylation E3 ligase, collaborating with the neddylation conjugating enzyme UBE2F to attach NEDD8 to Lys724 of CUL5. In contrast, the COP9 signalosome (CSN) complex removes cullin neddylation through a process known as deneddylation. The precise regulation of CUL5 neddylation is crucial for controlling CRL5‐associated biological processes, such as apoptosis, viral infection, proliferation, and migration. For instance, phosphorylation of CUL5 at Ser730 by protein kinase A (PKA) inhibits CUL5 neddylation and suppresses cell proliferation.^[^ ^18^ ^]^ Therefore, identifying the regulators of CUL5 neddylation is of significant interest.

The APC/C is the most complex E3 ubiquitin ligase in humans, consisting of at least 14 distinct subunits and one of two co‐activator subunits. The ligase core is composed of the RING finger protein APC11 and a cullin‐like subunit, APC2, which together facilitate the ubiquitylation of APC/C substrates.^[^ ^19^ ^]^ The co‐activator proteins CDC20 and CDH1 bind to APC/C at different stages of the cell cycle to recognize specific substrates and regulate cell cycle progression.^[^ ^13^ ^]^ Specifically, APC/C^CDC20^ is activated during early mitosis, where it degrades prometaphase substrates such as Cyclin A, followed by the degradation of Cyclin B and securin to facilitate the transition from metaphase to anaphase. APC/C^CDH1^, on the other hand, functions during anaphase and the G1 phase to degrade remaining mitotic regulators, including CDC20. As a key regulator of multiple cell cycle events, APC/C dysfunction is associated with various human diseases, particularly cancer. APC/C inhibitors, such as proTAME and apcin, can block mitotic exit and sensitize cancer cells to several anti‐cancer therapies, including microtubule‐inhibiting drug paclitaxel and the topoisomerase II inhibitor etoposide.^[^ ^20^, ^21^, ^22^ ^]^ Therefore, prolonging mitotic duration by inhibiting APC/C represents a promising anti‐tumor strategy to enhance treatment sensitivity.

In this study, we report a novel crosstalk between two multi‐subunit E3 ubiquitin ligases, CRL5 and APC/C, mediated by the direct interaction between CUL5 and APC11. Unlike its role as a RING protein in the APC/C E3 ligase complex, APC11 binds to and regulates CUL5 neddylation rather than facilitating substrate ubiquitylation. Biochemically, APC11 disruption promotes the binding of UBE2F to CUL5, resulting in excessive neddylation of CUL5 and subsequent destabilization of SOCS3. Thus, APC11, a well‐established cell cycle regulator, suppresses cell migration by stabilizing SOCS3 and promoting the degradation of its substrate, integrin β1. Meanwhile, the protein levels of APC11 are upregulated upon its binding to CUL5. Mechanistically, CUL5 recruits the ITCH E3 ligase to mediate K27, K29, and K33‐linked polyubiquitylation at Lys83 of APC11, thereby stabilizing it. CUL5 depletion impedes mitotic exit and enhances the chemosensitivity of cervical carcinoma HeLa cells to paclitaxel by downregulating APC11, in both in vitro cell cultures and in vivo xenograft tumor models. Therefore, maintaining the precise levels of both CUL5 and APC11 is critical for protein quality control, while deregulation of these proteins is linked to cancer metastasis and altered chemosensitivity.

## 2 Results

### 2.1 APC11 Directly Interacts with CUL5

**Figure 1:**
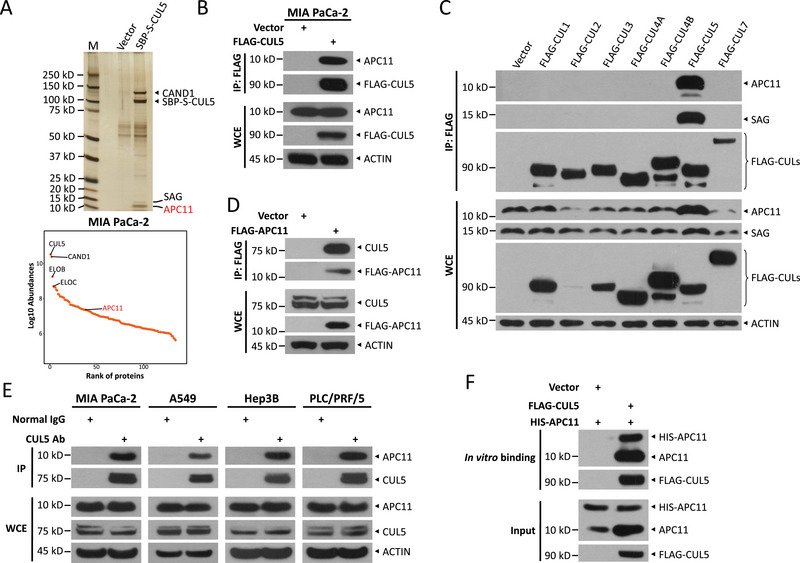
APC11 selectively interacts with CUL5 among cullin family members. a) Silver staining pattern of CUL5‐interacting proteins. MIA PaCa‐2 cells stably overexpressing SBP‐S‐CUL5 or mock vector pLVX were harvested for tandem affinity purification, followed by SDS‐PAGE and silver staining (top). APC11 was a candidate CUL5‐binding protein identified by LC‐MS/MS analysis (bottom, scatter plot of CUL5‐binding proteins). b) Co‐immunoprecipitaion (Co‐IP) of exogenous FLAG‐CUL5 and endogenous APC11 in MIA PaCa‐2 cells. c) Co‐IP of exogenous FLAG‐tagged cullin proteins and endogenous APC11 or SAG in HEK293 cells. d) The interaction of exogenous FLAG‐APC11 with endogenous CUL5 in HEK293 cells. e) Co‐IP of endogenous CUL5 and APC11 in MIA PaCa‐2, A549, Hep3B, and PLC/PRF/5 cells. f) CUL5 binds to APC11 in vitro. Recombinant HIS‐APC11 was incubated with purified FLAG‐CUL5 or vector in vitro, followed by FLAG‐pull‐down and then IB with anti‐APC11 and FLAG Abs. WCE, whole cell extracts.

CRL5 is critically involved in various biological processes, including signal transduction, viral infection, apoptosis, and migration. Its dysfunction has been implicated in several human diseases, particularly cancer.^[^ ^16^, ^17^ ^]^ To fully elucidate the role of CRL5 in physiological and pathological processes, we first generated pancreatic cancer MIA PaCa‐2 cells that stably express CUL5, tagged with a tandem streptavidin‐binding peptide (SBP) and an S tag. Subsequently, we performed tandem affinity purification (TAP) coupled with liquid chromatography‐tandem mass spectrometry (LC‐MS/MS) to identify the upstream regulators and novel downstream substrates of CRL5. Through this approach, we identified APC11, the RING protein of the APC/C E3 ubiquitin ligase, as a potential binding partner of CUL5 (**Figure** **1**a). Consistently, the BioGRID database (https://thebiogrid.org/) lists CUL5 as an interacting partner of APC11.^[^ ^23^, ^24^ ^]^ A subsequent co‐immunoprecipitation (Co‐IP) assay confirmed the interaction between FLAG‐tagged CUL5 and endogenous APC11 in MIA PaCa‐2 cells (Figure 1b). We further observed a selective binding of endogenous APC11 to ectopically expressed CUL5, but not other CULs in HEK293 cells (Figure 1c). In contrast, an inverse Co‐IP assay confirmed that endogenous CUL5 also binds to FLAG‐tagged APC11 (Figure 1d). Additionally, endogenous APC11 was readily detected in the immunoprecipitants of endogenous CUL5 from MIA PaCa‐2, A549, Hep3B, and PLC/PRF/5 cells (Figure 1e). Finally, an in vitro binding assay confirmed the direct interaction between purified FLAG‐CUL5 and recombinant HIS‐APC11 (Figure 1f). Collectively, these results establish that APC11 is a bona fide binding partner of CUL5.

### 2.2 APC11 Interacts with Non‐Neddylated CUL5 in a Distinct Manner, Unlike SAG

**Figure 2:**
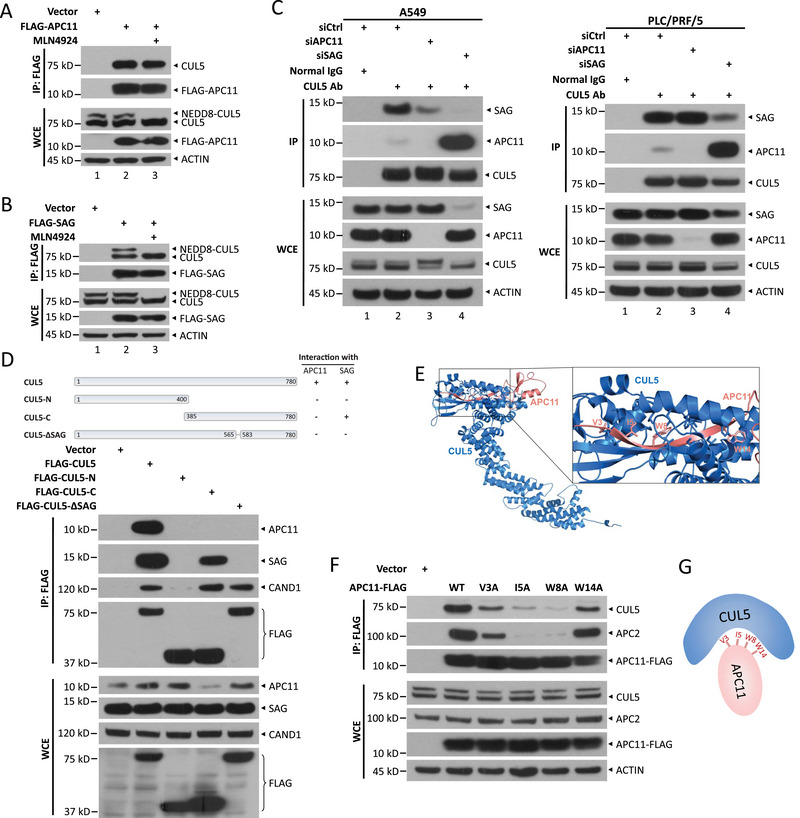
APC11 binds to CUL5 in a manner distinct from SAG. a,b) Co‐IP of transfected FLAG‐APC11 (a) or FLAG‐SAG (b) and endogenous CUL5 in HEK293 cells with 1 µM MLN4924 treatment for 12 h. c) The interaction of endogenous CUL5 with APC11 or SAG upon SAG or APC11 knockdown. A549 and PLC/PRF/5 cells were transfected with siRNA oligos targeting APC11, SAG or scrambled control siRNA for 72 h and then immunoprecipitated with IgG or anti‐CUL5 Ab. d) Co‐IP of endogenous APC11, SAG or CAND1 and FLAG‐tagged CUL5 truncations including wild‐type CUL5 (aa 1–780), CUL5‐N (aa 1–400), CUL5‐C (aa 385–780), and CUL5‐ΔSAG (deletion of aa 565‒582) in HEK293 cells. e) A structural modeling illustration of docking APC11 (red) to CUL5 (blue). The putative CUL5‐interaction sites in APC11 are shown in the enlarged image. f) Co‐IP of transfected FLAG‐APC11 wild‐type or mutants in N terminus (including V3A, I5A, W8A, and W14A) and endogenous CUL5 or APC2, in HEK293 cells. g) Schematic representation of the key APC11 residues involved in binding to full‐length CUL5.

CRL5 relies on RING protein SAG for ubiquitylation of its substrates, in contrast to other CRLs, which utilize RBX1.^[^ ^16^ ^]^ Given that APC11, a RING protein of the APC/C complex,^[^ ^19^ ^]^ shares functional similarities with SAG in CRL5 and selectively binds to CUL5, we hypothesized that APC11 might interact with CUL5 in a manner similar to SAG. Unexpectedly, we noticed that FLAG‐tagged APC11 preferentially interacted with non‐neddylated CUL5 (Figure 1d), whereas SAG, as the RING of an active CRL5 complex, undoubtedly binds to neddylated CUL5.^[^ ^25^ ^]^ To further investigate whether APC11 selectively binds to non‐neddylated CUL5, we used MLN4924, a small molecule inhibitor of NEDD8‐activating enzyme that inhibits cullin neddylation,^[^ ^15^ ^]^ in Co‐IP assays. Indeed, FLAG‐tagged APC11 selectively interacted with non‐neddylated CUL5, as indicated by the unchanged band size of CUL5 in immunoprecipitants regardless of MLN4924 treatment (**Figure** **2**a, lanes 3 vs 2). In contrast, FLAG‐tagged SAG bound to both neddylated and non‐neddylated forms of CUL5 (Figure 2b, lanes 3 vs 2). To avoid siRNA off‐target effects, all siRNA‐mediated knockdowns in this study were performed using two distinct siRNA oligonucleotides (oligos); this approach is applied consistently but not repeatedly emphasized in the following text. Moreover, silencing APC11 reduced or had no effect on the interaction between endogenous CUL5 and SAG in A549 and PLC/PRF/5 cells, (Figure 2c; Figure S1, Supporting Information, lanes 3 vs 2), indicating that APC11 does not compete with SAG for CUL5 binding. Interestingly, SAG silencing markedly enhanced the interaction between endogenous CUL5 and APC11 in A549 and PLC/PRF/5 cells (Figure 2c; Figure S1, Supporting Information, lanes 4 vs 2). Finally, we conducted Co‐IP assays to map the APC11 binding site on CUL5 by transiently transfecting three CUL5 truncation mutants into HEK293 cells: the N‐terminus (aa 1–400), the C‐terminus (aa 385–780), and a mutant lacking SAG binding site (deletion of aa 565–582), as well as full‐length CUL5 (aa 1–780). The results revealed that only full‐length CUL5 interacted with endogenous APC11, while SAG bound to the C‐terminus of CUL5 via amino acid 565–582 (Figure 2d). Collectively, these findings indicate that APC11 specifically interacts with full‐length, non‐neddylated CUL5, in a manner differently from SAG.

To further characterize the CUL5‐APC11 interaction, we performed molecular docking simulations to identify the key residues on APC11 involved in this binding. Our analysis revealed that four residues (V3, I5, W8, and W14) in the N‐terminus of APC11 are potential candidates for CUL5 binding (Figure 2e). We then generated four mutants, each with one of these residues replaced by alanine, and conducted Co‐IP assays in HEK293 cells. The W14A mutant significantly reduced the interaction between APC11 and CUL5, without much affecting the APC11‐APC2 binding. In contrast, the V3A, I5A, and W8A mutants nearly lost their ability to bind CUL5 or APC2 (Figure 2f). Thus, the W14 is critical for the interaction between APC11 and CUL5, but dispensable for the assembly of the APC/C complex. We then selected this W14 mutant for subsequent studies to investigate the biological outcomes of the APC11‐CUL5 interaction. These results demonstrate that four N‐terminal residues of APC11 (V3, I5, W8, and W14) are critical for its interaction with full‐length CUL5, whereas W14 is dispensable for its interaction with APC2 (Figure 2g).

### 2.3 APC11 Regulates CUL5 Neddylation and Substrate Degradation

**Figure 3:**
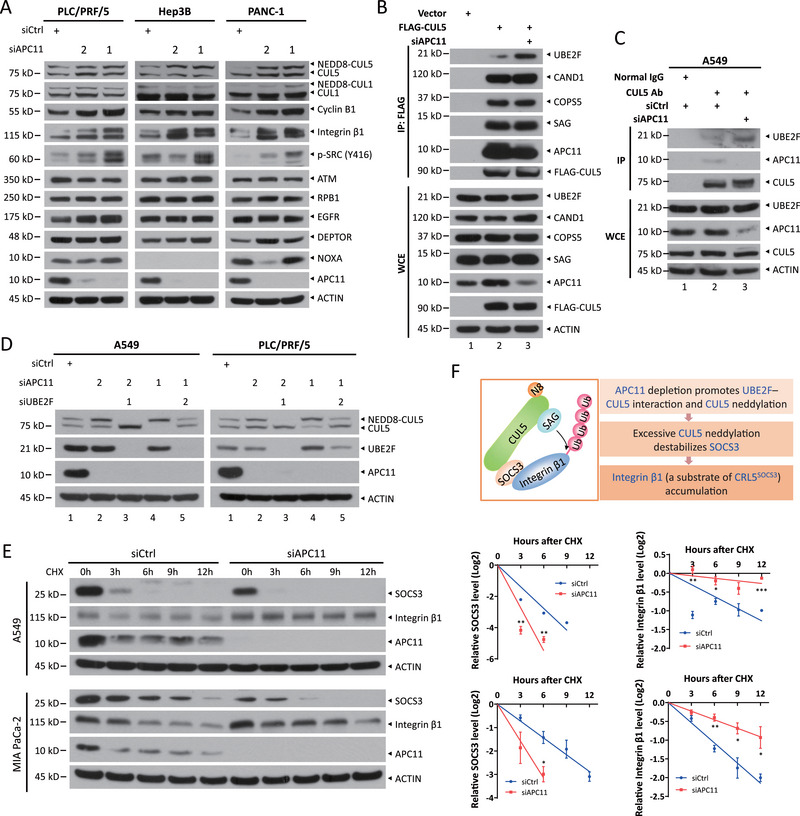
APC11 depletion promotes CUL5 neddylation but stabilizes integrin β1. a) Immunoblot of indicated proteins in PLC/PRF/5, Hep3B, and PANC‐1 cells transfected with siRNA targeting APC11 or with a scrambled control siRNA. b) Co‐IP of transfected FLAG‐CUL5 and endogenous CAND1, COPS5, UBE2F or SAG in HEK293 cells transfected with indicated siRNA oligos. c) Co‐IP of endogenous CUL5 with UBE2F or APC11 in A549 cells transfected with indicated siRNA oligos. d) Immunoblot of CUL5 in A549 and PLC/PRF/5 cells transfected with indicated siRNA oligos. e) The stability of SOCS3 and integrin β1 in A549 and MIA PaCa‐2 cells upon APC11 knockdown. A549 and MIA PaCa‐2 cells were transfected with indicated siRNA oligos for 72 h and then treated with 100 µg mL^−1^ CHX for the indicated time periods, followed by IB analysis. Densitometry quantifications were performed with ImageJ, and the decay curves are shown (right). Data are presented as mean ± SEM, *n* = 3. For statistical analysis, significances were determined by Student's *t*‐test. * *p* < 0.05, ** *p* < 0.01, *** *p* < 0.001. f) Diagram illustrating how APC11 depletion promotes CUL5 neddylation and leads to integrin β1 accumulation.

Next, we investigated whether APC11 would affect CRL5 activity through direct interaction with CUL5. Upon APC11 knockdown using two distinct siRNA oligos, we observed that the neddylation levels of CUL5, but not CUL1, were upregulated in multiple cancer cell lines, including PLC/PRF/5, Hep3B, PANC‐1, and A549 cells (**Figure** **3**a; Figure S2a, Supporting Information), along with the accumulation of Cyclin B1 or securin, well‐known substrates of APC/C complex, serving as a positive control for APC11 knockdown (Figure 3a; Figure S2a, Supporting Information). Additionally, silencing APC11 did not significantly affect CUL5 mRNA expression (Figure S2b, Supporting Information). Several key regulators are known to modulate CUL5 neddylation, including CAND1, the neddylation E2 UBE2F, the neddylation E3 SAG, and CSN complex.^[^ ^17^ ^]^ We found that APC11 knockdown had no effects on the levels of these regulators in A549 and Hep3B cells (Figure S2c, Supporting Information), suggesting that APC11 may influence CUL5 neddylation by modulating its interaction with these regulators. Indeed, silencing APC11 increased the binding of FLAG‐tagged CUL5 to endogenous UBE2F, without affecting CUL5 binding with CAND1, SAG or COPS5 (Figure 3b; Figure S2d, Supporting Information, lanes 3 vs 2). Likewise, APC11 knockdown promoted the interaction between endogenous CUL5 and UBE2F in A549 cells (Figure 3c; Figure S2e, Supporting Information, lanes 3 vs 2). Notably, simultaneous knockdown of UBE2F and APC11 completely rescued the increased levels of CUL5 neddylation triggered by APC11 knockdown in both A549 and PLC/PRF/5 cells (Figure 3d, lanes 3 vs 2 and 5 vs 4). Thus, APC11 affects CUL5 neddylation through UBE2F, a known E2 which partners with SAG to neddylate CUL5 likely by facilitating the binding between UBE2F and CUL5.

Neddylated CUL5 is considered the active form of CUL5, which activates CRL5 ligase to promote the degradation of its substrates.^[^ ^26^, ^27^ ^]^ Unexpectedly, we observed an accumulation of integrin β1, a substrate of the CUL5‐SOCS3 E3 ligase,^[^ ^28^ ^]^ in APC11 knockdown cells, along with elevated CUL5 neddylation (Figure 3a; Figure S2a, Supporting Information), without a corresponding increase in integrin β1 mRNA levels (Figure S2f, Supporting Information), suggesting a non‐transcriptional mechanism of regulation. Interestingly, among several well‐established CRL5 substrates that were examined, including integrin β1, p‐SRC (Y416), ATM, RPB1, EGFR, DEPTOR, and NOXA,^[^ ^16^ ^]^ only integrin β1 consistently accumulated across all cancer cell lines upon APC11 knockdown using two independent siRNAs targeting APC11 (Figure 3a). Notably, p‐SRC (Y416) also showed marked accumulation in most cases (Figure 3a). Previous studies have shown that the balance between neddylation and deneddylation of cullins regulates the stability of CRL components. Neddylated CULs are unstable, and deficiency in CSN activity leads to a decrease in the protein levels of CUL1 and CUL3.^[^ ^29^ ^]^ Moreover, CSN facilitates the function of CRLs by protecting their adapters, such as Skp1 and Btb3p, from autocatalytic destruction.^[^ ^30^ ^]^ Given the reduction of SOCS3 protein levels upon APC11 knockdown in A549 cells (Figure S2a, Supporting Information), we next investigated whether excessive CUL5 neddylation would destabilize its receptor SOCS3, resulting in integrin β1 accumulation. Using cycloheximide (CHX) to block new protein synthesis, we found that silencing APC11 shortened the protein half‐life of SOCS3, but extended the protein half‐life of integrin β1 in A549, MIA PaCa‐2, and PANC‐1 cells (Figure 3e; Figure S2g,h, Supporting Information). Consistently, silencing COPS5 to inactivate CSN increased the neddylation levels of CUL1 and CUL5, reduced the levels of receptor subunits (such as FBXW7, SKP2, and SOCS3), and led to the accumulation of substrates like c‐JUN (a substrate of SCF^FBXW7^), p27 (a substrate of SCF^SKP2^), and integrin β1 (a substrate of CRL5^SOCS3^) (Figure S2i, Supporting Information, Supporting Information). Together, these findings demonstrate that APC11 regulates CUL5 neddylation by inhibiting the interaction between UBE2F and CUL5, and its knockdown enhanced CUL5 neddylation to destabilize SOCS3, leading to integrin β1 accumulation (Figure 3f).

### 2.4 APC11 Silencing Promotes Cell Migration and Tumor Metastasis by Inhibiting the Degradation of Integrin β1

**Figure 4:**
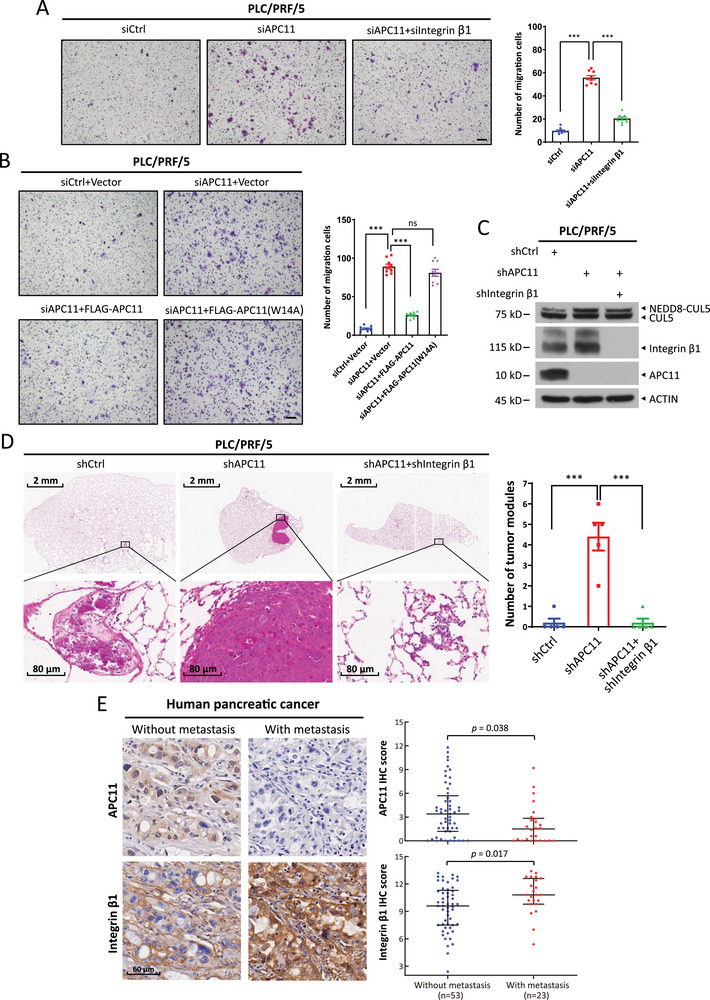
APC11 silencing facilitates cell migration and tumor metastasis by suppressing integrin β1 degradation. a,b) PLC/PRF/5 cells were transfected with indicated siRNA oligos or plasmids for 48 h, followed by a transwell cell migration assay. The wild‐type and W14A‐mutant FLAG‐APC11 plasmids were modified to include synonymous mutations at the siAPC11 target site (b). Representative migration images from one out of three biological replicates are shown (a and b, left). Scale bar, 100 µm. The number of migration cells was counted in three random fields per chamber (a and b, right). Data are shown as mean ± SEM, *n* = 3. c,d) PLC/PRF/5 cells were infected with indicated lentiviral shRNA viruses and then injected into nude mice via tail vein. Immunoblot of indicated proteins in the stable cells (c) and representative H&E staining of lung lobe sections from nude mice injected with indicated stable cells (d, left) are shown. Scale bars, 2 mm (top), 80 µm (bottom). The number of lung metastasis tumors in all five lung lobes from each mouse was counted (d, left). Data are presented as mean ± SEM, *n* = 5. For statistical analysis, significances were determined by Student's *t*‐test. *** *p* < 0.001, ns, not significant. e) Human pancreatic cancer tissue microarrays, comprising 23 metastatic (lymph node or distant) and 53 non‐metastatic pancreatic cancer samples, were immunostained for APC11 and integrin β1. Representative images of APC11 and integrin β1 staining in metastatic and non‐metastatic tissues are shown (scale bar, 60 µm; left). The staining quantification was determined by IHC scoring using the IRS system, based on staining intensity and the percentage of positive cells (right). Statistical significance was assessed using the Wilcoxon rank‐sum test.

A previous study has shown that the CRL5^SOCS3^ E3 ligase inhibits metastasis of small‐cell lung cancer cells by degrading integrin β1.^[^ ^28^ ^]^ To determine whether APC11 regulates cell migration through interaction with CUL5, we first conducted transwell assays in PLC/PRF/5 and A549 cells, and found that silencing APC11 enhanced cell migration, an effect that was largely abrogated by simultaneous knockdown of integrin β1 (**Figure** **4**a; Figure S3a,b, Supporting Information), suggesting a causal role of integrin β1. However, knockdown of APC2 had no such effect on the migration of PLC/PRF/5 cells (Figure S3c, Supporting Information), excluding possible involvement of the APC/C complex in APC11‐regulated cell migration. Furthermore, ectopic expression of wild‐type APC11, but not the W14A mutant (which reduces APC11 binding to CUL5), reversed the migration promoted by APC11 knockdown (Figure 4b; Figure S3d, Supporting Information). Moreover, we extended this observation to an in vivo metastasis model by injecting PLC/PRF/5 cells with stable APC11 knockdown alone or in combination with stable integrin β1 knockdown into the tail veins of nude mice. The silencing efficiency of APC11 and integrin β1 was verified by immunoblotting (Figure 4c). Six weeks post injection, the mice were euthanized, and the lungs—being the primary organ for metastasis following tail vein injection—were collected. Compared to the control groups, the APC11 knockdown group showed a significant increase in the number of lung metastasis nodules, an effect that was completely rescued by simultaneous knockdown of integrin β1 (Figure 4d). Furthermore, pancreatic tissues from *Kras^G12D^;Pdx1‐Cre+* mice, with or without liver metastasis at ≈15 months of age, were analyzed for APC11 and integrin β1 protein levels. Compared to tissues without liver metastasis, pancreatic tissues with liver metastasis exhibited reduced APC11 staining and increased integrin β1 staining (Figure S3e, Supporting Information). Additionally, although the difference was not statistically significant, pancreatic adenocarcinoma (PAAD) patients with lower APC11 mRNA levels tended to have a lower overall survival probability (Figure S3f, Supporting Information). Finally, we assessed APC11 and integrin β1 protein levels in human pancreatic cancer tissue microarrays using immunohistochemistry (IHC). APC11 level was significantly decreased, whereas integrin β1 level was elevated in metastatic tumors (*n* = 23, lymph node/distant) compared with non‐metastatic tumors (*n* = 53) (Figure 4e), suggesting their potential cooperative role in promoting pancreatic cancer progression and malignancy. Collectively, APC11 deficiency promotes cell migration in vitro and tumor metastasis in vivo by disrupting CUL5‐mediated degradation of integrin β1.

### 2.5 APC11 is Protected From Proteasomal Degradation Due to its Interaction with CUL5

**Figure 5:**
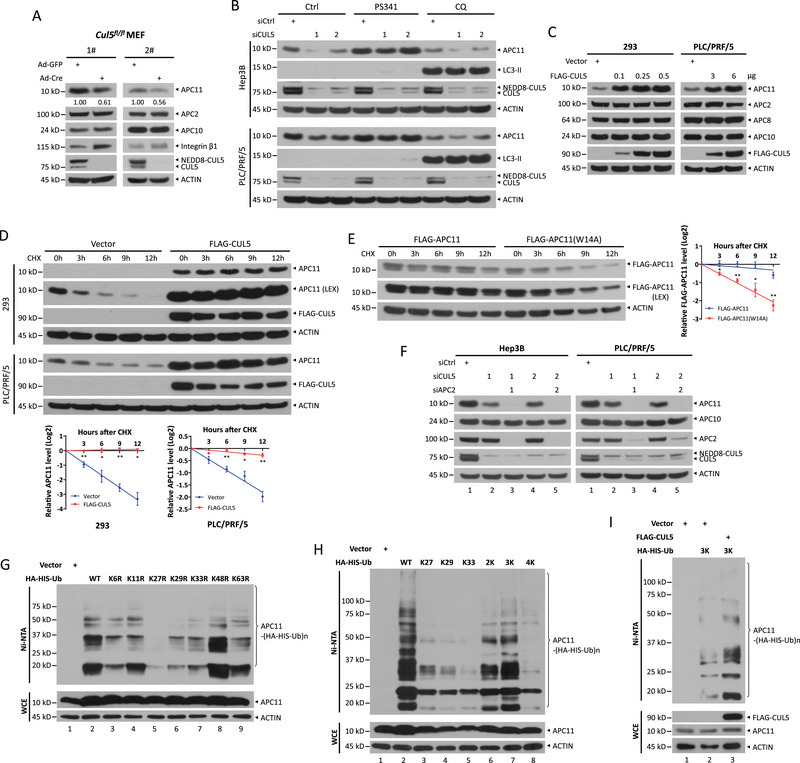
CUL5 protects APC11 from proteasomal degradation via adding K27, K29, and K33‐linked polyubiquitylation. a) Immunoblot of indicated proteins in primary *Cul5^fl/fl^* MEF cells following infection with Ad‐Cre or Ad‐GFP for 72 h. b) Immunoblot of APC11, LC3 and CUL5 in Hep3B and PLC/PRF/5 cells upon CUL5 knockdown by two distinct siRNA oligos, following treatment with PS341 (1 µM) or CQ (50 µM) for 12 h. c) Immunoblot of APC11 and other components of the APC/C complex in HEK293 and PLC/PRF/5 cells after transfection with various amounts of FLAG‐CUL5 for 48 h. d) The stability of APC11 in HEK293 and PLC/PRF/5 cells with overexpression of CUL5. HEK293 and PLC/PRF/5 cells were transfected with FLAG‐CUL5 plasmids for 48 h, and then treated with CHX (100 µg mL^−1^) for the indicated time periods, followed by IB analysis. e) Degradation of wild‐type APC11 and its W14A mutant in HEK293 cells. HEK293 cells were transfected with indicated plasmids for 48 h, and then treated with CHX (100 µg mL^−1^) for the indicated time periods, followed by IB analysis. Densitometry quantifications were performed with ImageJ, and the decay curves are shown (d, bottom and e, right). Data are presented as mean ± SEM, *n* = 3. For statistical analysis, significances were determined by Student's *t*‐test. * *p* < 0.05, ** *p* < 0.01. f) Immunoblot of indicated proteins in Hep3B and PLC/PRF/5 cells transfected with indicated siRNA oligos for 72 h. g,h) In vivo ubiquitylation assay to assess the type of ubiquitin chains covalently attached to APC11. HEK293 cells were transfected with indicated K→R mutants (g) or only the indicated lysine residue(s) mutants (2K, K27/K29; 3K, K27/K29/K33; 4K, K6/K11/K48/K63) (h) of ubiquitin for 48 h, followed by purification with Ni‐NTA under denaturing conditions. Pull‐downs (top) and WCE (bottom) were subjected to IB analysis with indicated Abs, respectively. i) K27/K29/K33‐linked ubiquitylation of APC11 promoted by overexpression of CUL5. HEK293 cells were co‐transfected with indicated plasmids, followed by purification with Ni‐NTA. Pull‐downs (top) and WCE (bottom) were subjected to IB with indicated Abs.

Given that APC11 interacts with CUL5 and regulates CUL5 neddylation, we wondered whether CUL5, in turn, also affects APC11. We first deleted CUL5 in two distinct primary *Cul5^fl/fl^* MEFs using adenovirus‐expressing Cre recombinase (Ad‐Cre) and observed that the protein levels of APC11 were specifically decreased in *Cul5*‐null cells, while other APC/C components (such as APC2 and APC10) remained unchanged. This decrease in APC11 levels was accompanied by the accumulation of integrin β1, a substrate of CRL5, as a positive control (**Figure** **5**a). Consistently, silencing CUL5 reduced APC11 levels in Hep3B and PLC/PRF/5 cells, and notably, the proteasome inhibitor PS341—but not autophagy inhibitor chloroquine (CQ)—restored APC11 levels (Figure 5b), indicating that CUL5 depletion promotes proteasomal degradation of APC11. Conversely, ectopic expression of FLAG‐tagged CUL5 increased APC11 protein levels in a dose‐dependent manner in HEK293, PLC/PRF/5, and Hep3B cells (Figure 5c; Figure S4a, Supporting Information). Furthermore, overexpressing the K724R mutant of CUL5, which is unable to undergo neddylation and thus remains inactive, also increased APC11 levels (Figure S4b, Supporting Information), suggesting that CUL5 stabilizes APC11 independently of its E3 ligase activity. Moreover, neither CUL5 knockdown, nor CUL5 overexpression had little, if any, effect on APC11 mRNA levels (Figure S4c,d, Supporting Information), ruling out the possible regulation at the transcriptional level. To further elucidate the role of CUL5 in modulating APC11 stability, we examined the half‐life of APC11 and found that overexpression of CUL5 significantly extended the protein half‐life of APC11 (Figure 5d), while CUL5 knockdown shortened its half‐life (Figure S4e, Supporting Information). Notably, the half‐life of the APC11 W14A mutant, which reduces APC11 binding to CUL5, was obviously shorter than that of the wild‐type protein (Figure 5e). These data demonstrate that CUL5 binds to and protects APC11 from proteasomal degradation.

### 2.6 APC11 is Stabilized by CUL5 via K27, K29, and K33‐Linked Polyubiquitylation

Next, we elucidated the mechanism by which CUL5 stabilizes APC11. A previous study has shown that unassembled APC11 is degraded by APC/C itself.^[^ ^31^ ^]^ We then silenced APC2 to inhibit APC/C activity and found that APC2 knockdown did not restore the APC11 levels in CUL5‐depleted cells (Figure 5f, lanes 3 vs 2, and 5 vs 4). Given that APC/C E3 ligases typically degrade substrates via K11‐linked ubiquitin chains,^[^ ^32^ ^]^ we conducted in vivo ubiquitylation assays to identify the ubiquitin linkages involved in CUL5‐mediated APC11 stabilization. We transfected a series of ubiquitin mutants, each with an individual lysine replaced by arginine, into HEK293 cells. We observed a significant reduction in APC11 polyubiquitylation when the residue of K27, K29, or K33 was mutated (Figure 5g, lanes 5‒7 vs 2), while K11R had minor, if any, effect on APC11 polyubiquitylation, suggesting that CUL5 regulates APC11 stability independently of the APC/C complex. To further characterize these atypical linkages, we constructed various ubiquitin mutants capable of forming ubiquitin linkages at a single lysine (K27, K29, or K33), or combinations of K27 and K29 (2K), K27, K29, and K33 (3K), or K6, K11, K48, and K63 (4K). The in vivo ubiquitylation assay revealed that APC11 polyubiquitylation primarily consisted of a mixture of K27, K29, and K33 linkages (Figure 5h, lanes 7 vs 2‒6 and 8). Moreover, overexpression of FLAG‐tagged CUL5 increased the K27, K29, and K33‐linked polyubiquitylation of APC11 (Figure 5i, lanes 3 vs 2), indicating that these linkages are involved in APC11 stabilization. Collectively, these data demonstrate that CUL5 promotes K27, K29, and K33‐linked polyubiquitylation of APC11, unexpectedly leading to its stabilization.

### 2.7 CUL5 Facilitates ITCH to add K27, K29, and K33‐Linked Polyubiquitin Chains to APC11 at Lys83

**Figure 6:**
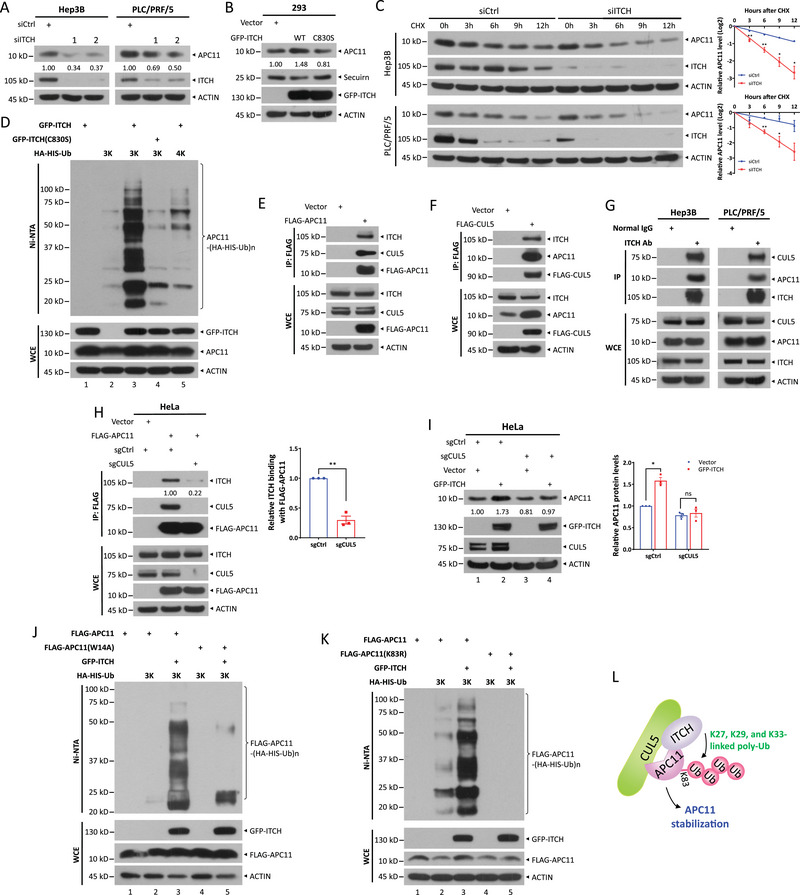
ITCH stabilizes APC11 by adding K27, K29, and K33‐linked polyubiquitin chains to APC11 at Lys83 in a CUL5‐dependent manner. a) Immunoblot of APC11 and ITCH in Hep3B and PLC/PRF/5 cells upon ITCH knockdown by two distinct siRNA oligos. b) Immunoblot of APC11 and APC/C substrate securin in HEK293 cells after transfection with wild‐type ITCH or its C830S mutant for 48 h. c) The stability of APC11 in Hep3B and PLC/PRF/5 cells upon ITCH knockdown. Hep3B and PLC/PRF/5 cells were transfected with indicated siRNA oligos for 72 h and then treated with CHX (100 µg mL^−1^) for the indicated time periods, followed by IB analysis. Densitometry quantifications were performed with ImageJ, and the decay curves are shown (right). d) K27/K29/K33‐linked ubiquitylation of APC11 promoted by overexpression of ITCH but not its C830S mutant. HEK293 cells were co‐transfected with indicated plasmids, followed by purification with Ni‐NTA. Pull‐downs (top) and WCE (bottom) were subjected to IB with indicated Abs. e) Co‐IP of exogenous FLAG‐tagged APC11 with endogenous ITCH or CUL5 in HEK293 cells. f) The interaction of exogenous FLAG‐tagged CUL5 with endogenous ITCH or APC11 in HEK293 cells. g) Co‐IP of endogenous ITCH with CUL5 or APC11 in Hep3B and PLC/PRF/5 cells. h) Co‐IP of exogenous FLAG‐tagged APC11 in HeLa cells upon CUL5 knockout mediated by CRISPR‐Cas9. HeLa cells with or without CUL5 were infected with lentivirus expressing FLAG‐APC11 for 72 h, and then harvested for IP analysis. Band intensities were quantified using ImageJ, and the relative ITCH‐APC11 binding affinity in sgCUL5 cells was compared to sgCtrl cells. i) Immunoblot of APC11 in sgCtrl or sgCUL5 HeLa cells after transfection with GFP‐ITCH for 48 h. Band intensities were quantified using ImageJ and expressed as relative gray values (normalized to sgCtrl/Vector cells), shown beneath each band. j,k) ITCH facilitates K27/K29/K33‐linked ubiquitylation of wild‐type APC11 but not its W14A (j) or K83R (k) mutant. HEK293 cells were co‐transfected with indicated plasmids, followed by purification with Ni‐NTA. Pull‐downs (top) and WCE (bottom) were subjected to IB with indicated Abs. Data are presented as mean ± SEM, *n* = 3 (c, h, i; right). For statistical analysis, significances were determined by Student's *t*‐test.* *p* < 0.05, ** *p* < 0.01, ns, not significant. l A model illustrating that CUL5 stabilizes APC11 through ITCH‐mediated K27/K29/K33‐linked polyubiquitylation at Lys83.

To define the E3 ligase responsible for the K27/K29/K33‐polyubiquitylation of APC11, we first tested the involvement of the CRLs, the largest E3 ligase family. However, treatment with MLN4924, which inhibits CULs neddylation, did not affect APC11 protein levels, even at high dose (Figure S5a, Supporting Information), suggesting that APC11 is not a substrate of CRLs. Several E3 ligases, including WSB1,^[^ ^33^ ^]^ ITCH,^[^ ^34^, ^35^, ^36^ ^]^ AREL1,^[^ ^37^ ^]^ TRIP12,^[^ ^37^ ^]^ and TRAF4,^[^ ^38^ ^]^ are known to modulate atypical K27, K29, and K33‐linked polyubiquitylation. We compared the effects of knockdown of these E3 ligases on APC11 levels and found that only silencing ITCH decreased APC11 levels (Figure S5b, Supporting Information). Notably, among these five E3 ligases, ITCH is the only one predicted by UbiBrowser (http://ubibrowser.ncpsb.org.cn).^[^ ^39^ ^]^ The impact of ITCH on APC11 stability was further verified using two independent siRNA oligos targeting ITCH in Hep3B and PLC/PRF/5 cells (**Figure** **6**a). Meanwhile, ectopic expression of wild‐type ITCH, but not the catalytically inactive mutant (C830S), increased APC11 levels, accompanied by a decrease in securin levels (Figure 6b). Moreover, ITCH silencing shortened the protein half‐life of APC11 in both Hep3B and PLC/PRF/5 cells (Figure 6c; Figure S5c, Supporting Information). The in vivo ubiquitylation assay showed that overexpressing wild‐type ITCH, but not the C830S mutant, dramatically promoted K27/K29/K33‐linked polyubiquitylation of APC11 (Figure 6d, lanes 4 vs 3). In contrast, wild‐type ITCH had minimal, if any, effect on K6/K11/K48//K63‐linked polyubiquitylation of APC11 (Figure 6d, lanes 5 vs 2). These results demonstrate that ITCH stabilizes APC11 by adding K27/K29/K33‐linked polyubiquitin chains on APC11.

Considering the role of CUL5 in stimulating K27/K29/K33‐linked polyubiquitylation of APC11 (Figure 5i), we next examined whether CUL5 is involved in ITCH‐mediated APC11 ubiquitylation. We found that FLAG‐tagged APC11 pulled down endogenous ITCH and CUL5 simultaneously (Figure 6e), and similarly, FLAG‐tagged CUL5 precipitated with endogenous ITCH and APC11 concurrently (Figure 6f). Furthermore, endogenous APC11 and CUL5 were readily detected in the immunoprecipitants of endogenous ITCH from both Hep3B and PLC/PRF/5 cells (Figure 6g), suggesting that APC11, CUL5, and ITCH form a ternary complex. However, in CUL5‐null HeLa cells, the interaction between FLAG‐tagged APC11 and ITCH was abolished (Figure 6h). Consistently, ectopic expression of wild‐type ITCH increased APC11 levels in HeLa cells, but not in CUL5 knockout HeLa cells (Figure 6i, lanes 3‒4 vs 1‒2), indicating a causal role of CUL5 for ITCH‐mediated APC11 accumulation. Indeed, ITCH promoted the K27/K29/K33‐linked polyubiquitylation of wild‐type APC11, but not the W14A mutant, which significantly reduces its binding to CUL5 (Figure 6j, lanes 5 vs 3).

Finally, we proceeded to identify the specific lysine residue(s) on APC11 conjugated with the K27/K29/K33‐linked polyubiquitin chains. There are seven lysine residues on APC11, and we generated individual lysine‐to‐arginine mutants of APC11 for each residue. We found that the K83R mutant showed significantly reduced levels compared to the wild‐type APC11, and K83R levels were completely recovered with MG132 treatment (Figure S5d,e, Supporting Information). Overexpression of ITCH increased the K27/K29/K33‐linked polyubiquitylation of wild‐type APC11, but had no effect on that of the K83R mutant (Figure 6k, lanes 5 vs 3). Consistent with these findings, BioGRID data indicate Lys83 as the predominant ubiquitylation site of APC11.^[^ ^40^, ^41^ ^]^ Together, these results demonstrate that ITCH stabilizes APC11 by attaching K27/K29/K33‐linked polyubiquitin chains to Lys83 in a CUL5‐dependent manner (Figure 6l).

### 2.8 CUL5 Disruption Impairs Mitotic Exit and Sensitizes Cells to Paclitaxel

**Figure 7:**
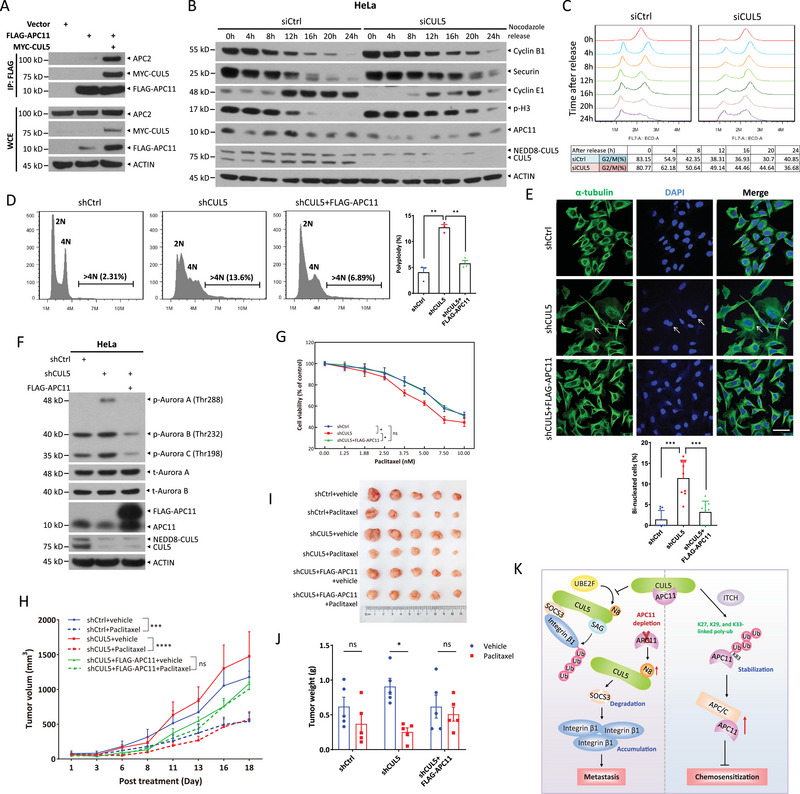
CUL5 depletion induces polyploidy and sensitizes cells to paclitaxel. a) Co‐IP of FLAG‐tagged APC11 and endogenous APC2 in HEK293 cells with or without co‐transfection of MYC‐tagged CUL5. b,c) HeLa cells were transfected with siRNA oligos targeting CUL5 or with a scrambled control siRNA for 48 h, and then synchronized in M phase by 100 ng mL^−1^ nocodazole treatment for 18 h. Next, the cells released into the cell cycle for different time periods, followed by IB with indicated Abs (b) or FACS analysis (c). d–g) HeLa cells were infected with lentiviral viruses expressing shRNA targeting CUL5 or ectopic expressing FLAG‐tagged APC11 and then selected with puromycin. Cell cycle profiles of the stable cells determined by FACS are shown and the polyploidy population with >4N DNA content are marked (d, left) (mean ± SEM, *n* = 3). Immunofluorescence analysis of α‐tubulin and DAPI in the stable cells, the representative images of binucleated cells indicated with arrows are shown (scale bar, 50 µm) (e, top), and the percentage of binucleated cells were counted from 9 random fields (mean ± SD) (e, bottom). Immunoblot of indicated proteins in the stable HeLa cells (f). The CCK8 assays were employed to assess the sensitivity of the stable HeLa cells to paclitaxel treatment (mean ± SEM) (g). h–j) One million of indicated stable HeLa cells were injected subcutaneously into both flanks of BALB/c nude mice. After 5 days, paclitaxel (15 mg kg^−1^) or vehicle was intraperitoneally injected three times a week. 18 days post‐treatment, tumor growth curve (h), tumor picture (i), and tumor weight (j) of tumors from indicated groups are shown. Data are presented as mean ± SEM, *n* = 5. For statistical analysis, significances were determined by Student's *t*‐test.* *p* < 0.05, ** *p* < 0.01, *** *p* < 0.001, **** *p* < 0.0001, ns, not significant. k) A working model for the crosstalk between CRL5 and APC/C E3 ligase in the regulation of cancer metastasis and chemosensitivity. See text for details.

We next investigated whether upregulated APC11 upon CUL5 overexpression facilitates APC/C complex formation. The Co‐IP assays showed that ectopic expression of MYC‐tagged CUL5 dramatically enhanced the interaction between FLAG‐tagged APC11 and endogenous APC2 (**Figure** **7**a), suggesting that upregulated APC11 promotes the assembly of additional APC/C complexes. Considering the critical role of APC/C in cell cycle progression, we hypothesized that CUL5 might modulate APC/C‐dependent biological processes by stabilizing APC11. To test this, we synchronized cells at the G2/M phase using nocodazole, and then released them into the cell cycle to assess the role of CUL5 in mitotic exit. Immunoblotting revealed that Cyclin B1 and securin—APC/C substrates degraded during M phase—were largely diminished by 16 h post‐release in control cells, whereas in CUL5 knockdown HeLa cells, their degradation was delayed until 24 h. Consistently, the mitotic marker phospho‐histone H3 (p‐H3) declined markedly by 12 h in control cells, but persisted until 20 h in CUL5‐depleted cells. Cyclin E1, a G1/S phase marker, was increased dramatically by 12 h in controls, but showed only a modest rise in CUL5 knockdown cells (Figure 7b). These findings suggest that CUL5 silencing impairs mitotic exit. Meanwhile, flow cytometry analysis confirmed that CUL5 knockdown cells exited the G2/M phase more slowly than control cells (Figure 7c). A comparable delay in mitotic exit was observed in HeLa cells following CUL5 knockdown with distinct siRNA oligos (Figure S6a,b, Supporting Information), and in Hep3B cells depleted of CUL5 using two independent siRNA oligos (Figure S6c, Supporting Information).

To further examine the role of CUL5 in regulating APC/C activity in vivo, we generated stable HeLa cell lines with CUL5 knockdown, either alone or combined with APC11 stable overexpression. Flow cytometry revealed that CUL5 knockdown caused a 3‐fold increase in the polyploid cell population (>4N DNA content), a phenotype largely rescued by APC11 overexpression (Figure 7d). Immunofluorescence confirmed that CUL5 knockdown significantly increased the proportion of bi‐nucleated cells (Figure 7e, arrows), which was similarly dependent on APC11 downregulation. Furthermore, the phosphorylation of Aurora A/B/C—key regulators of centromere separation and microtubule dynamics—was elevated upon CUL5 knockdown, which was abrogated by simultaneous APC11 overexpression (Figure 7f). Collectively, these results indicate that CUL5 knockdown reduces APC11 levels, thereby impairing mitotic exit and promoting polyploidy.

Given that cancer cells with weak mitotic checkpoints or rapid slippage are often resistant to microtubule‐targeting drugs, strategies that delay mitotic exit may enhance the efficacy of such drugs.^[^ ^42^ ^]^ In line with this, previous studies have shown that the APC/C inhibitor proTAME sensitizes cancer cells to paclitaxel by inhibiting mitotic exit.^[^ ^21^ ^]^ Consistently, we found that knockdown of APC2 or APC11, both essential APC/C components, sensitized HeLa cells to paclitaxel (Figure S6d, Supporting Information). We next tested whether CUL5 depletion‐induced destabilization of APC11 would enhance cell sensitivity to paclitaxel. Indeed, HeLa cells with CUL5 knockdown exhibited increased paclitaxel sensitivity, and this effect was fully reversed by APC11 overexpression (Figure 7g). Finally, in an in vivo xenograft tumor model, CUL5 knockdown significantly enhanced the tumor‐suppressive effect of paclitaxel, as reflected in reduced tumor volume and weight. However, this enhanced response was abolished when APC11 was concurrently overexpressed (Figure 7h–j). These findings indicate that CUL5 depletion sensitizes cancer cells to paclitaxel through downregulation of APC11.

## 3 Discussion

The CRL and APC/C complexes are two major E3 ubiquitin ligases that play critical roles in regulating cell cycle progression.^[^ ^43^ ^]^ These complexes exhibit antagonistic crosstalk by targeting each other's components for degradation. For example, the SCF (SKP1‐CUL1‐F‐box) complex, also known as CRL1, couples with its receptor β‐TrCP to ubiquitylate and degrade CDH1 during the S phase.^[^ ^44^ ^]^ Conversely, in the G1 phase, APC/C^CDH1^ targets the F‐box protein SKP2 for degradation, leading to the accumulation of p21 and p27—substrates of SCF^SKP2^—and thereby maintaining the G1 phase.^[^ ^45^ ^]^ In addition, EMI1 (also known as FBXO5), a pseudo‐substrate of APC/C, inhibits APC/C activity by competitively binding to its core components and co‐activators CDH1 and CDC20, thereby blocking access of true substrates.^[^ ^46^ ^]^ Notably, inhibition of CSN with the small molecule CSN5i‐3 induces FBXO5 autodegradation, which in turn activates APC/C^CDH1^ and substantially contributes to the toxicity associated with CSN inhibition.^[^ ^47^ ^]^ Furthermore, a recent study demonstrated that SAG competes with APC2 for binding to the E2 enzymes UBE2C and UBE2S, thereby inhibiting APC/C functions.^[^ ^48^ ^]^ In this study, we uncovered a novel crosstalk mechanism between the CRL5 and APC/C complexes through a direct interaction between their respective components, CUL5 and APC11. Notably, APC11 specifically interacts with CUL5, but not other cullin family members (Figure 1c). CRL5 is particularly unique among CRLs due to several distinguishing features: (1) it utilizes SAG as its RING protein, whereas other CRLs use RBX1;^[^ ^16^ ^]^ (2) UBE2F serves as the E2 for CUL5 neddylation, while UBE2M performs this role for other cullins;^[^ ^17^ ^]^ and (3) the RBR‐type E3 ligase ARIH2 associates with CRL5, whereas ARIH1 acts in concert with other CRLs.^[^ ^49^, ^50^ ^]^ However, the mechanisms underlying these unique features of CUL5 remain largely unknown. Further investigation may help unravel why APC11 specifically binds to CUL5, shedding light on the functional uniqueness of CRL5.

As the RING component of the APC/C complex, APC11 binds to CUL5 in a manner distinct from that of the canonical RING protein, SAG (Figure 2; Figure S1, Supporting Information). First, SAG interacts with both neddylated and non‐neddylated forms of CUL5, whereas APC11 preferentially binds to the non‐neddylated form. Second, if APC11 functioned as a RING protein for CRL5 similar to SAG, it would be expected to compete with SAG for CUL5 binding. However, silencing APC11 either had no effect or reduced the interaction between CUL5 and SAG, rather than enhancing it. Third, CUL5 binds to SAG through a specific C‐terminal region encompassing amino acids 565–582, whereas APC11 requires the full‐length CUL5 for effective binding. These observations suggest that APC11 interacts with CUL5 in a unique, non‐canonical manner, which imparts an unexpected regulatory role in CRL5 function. Specifically, silencing APC11 selectively increased the neddylation of CUL5—but not CUL1—by enhancing the interaction between CUL5 and UBE2F (Figure 3; Figure S2, Supporting Information). Neddylation of CUL5 induces a conformational change at the CUL5‐SAG interface, thereby activating the CRL5 complex.^[^ ^51^ ^]^ While several enzymes involved in CUL5 neddylation and deneddylation have been identified, other regulatory mechanisms remain largely uncharacterized. To date, it has been reported that PKA phosphorylates CUL5 at Ser730, thereby inhibiting its neddylation at Lys724,^[^ ^18^ ^]^ and that PRDX1 oligomers form a ternary complex with UBE2F and CUL5 to promote its neddylation.^[^ ^52^ ^]^ Our findings reveal a novel mechanism whereby APC11 suppresses CUL5 neddylation by inhibiting its interaction with UBE2F.

Interestingly, APC11 knockdown‐induced CUL5 neddylation did not lead to enhanced degradation of CRL5 substrates, as might be expected. Instead, it resulted in the accumulation of integrin β1. Mechanistically, excessive neddylation of CUL5 destabilized its receptor protein SOCS3, shortening SOCS3's half‐life and thereby prolonging that of integrin β1 (Figure 3e; Figure S2g,h, Supporting Information). Our findings are consistent with previous studies showing that optimal neddylation levels are essential for maintaining the stability of CRL components, including CULs, their adaptors, and receptors.^[^ ^29^, ^30^, ^47^ ^]^ Disruption of this balance can impair associated biological processes. In this study, we further discovered that APC11, beyond its classical role in regulating cell cycle progression via the APC/C complex, also modulates cancer cell migration in vitro and metastasis in vivo (Figure 4; Figure S3, Supporting Information). APC11 silencing promoted cancer cell migration through a mechanism dependent on its interaction with CUL5 and the resulting accumulation of integrin β1. Simultaneous knockdown of integrin β1 significantly reduced the number of lung metastasis nodules induced by APC11 silencing. Consistently, decreased APC11 levels accompanied by elevated integrin β1 levels were observed in human pancreatic cancer tissues with lymph node or distant metastasis. These findings suggest that while APC11‐targeting compounds may be promising for modulating cell cycle events, their potential to promote cancer metastasis should be carefully considered prior to their application.

As a component of the CRL complex, cullins typically stabilize their associated RING proteins, such as RBX1 and SAG, by preventing their proteasomal degradation through direct binding—an expected function.^[^ ^53^ ^]^ Interestingly, CUL5 also stabilizes APC11 through a similar binding mechanism, revealing a previously unrecognized regulatory interaction (Figure 5; Figure S4, Supporting Information). Silencing CUL5 decreased the protein levels of APC11, an effect that was reversed by the proteasome inhibitor PS341 but not by lysosome inhibitor CQ, indicating that CUL5 protects APC11 from proteasomal degradation as well. More importantly and intriguingly, CUL5 stabilizes APC11 by promoting the formation of atypical K27/K29/K33‐linked polyubiquitin chains on APC11. The assembly of specific ubiquitin linkages is orchestrated by particular combinations of E2 and E3 enzymes. However, with ≈40 E2s and over 600 E3s encoded in the human genome, the potential for thousands of E2‐E3 combinations complicates the identification of mechanisms responsible for specific chain types.^[^ ^5^ ^]^ Currently, it is well established that the APC/C and SAG E3 ligases collaborate with UBE2C and UBE2S to generate K11‐linked polyubiquitin chains, whereas SCF complexes cooperate with UbcH5a and CDC34 to assemble K48‐linked chains.^[^ ^32^, ^48^, ^54^, ^55^ ^]^ However, the E2 enzymes responsible for catalyzing K27‐, K29‐, or K33‐linked chains remain unknown. Recent studies have suggested that HECT‐type E3 ligases are key modulators of atypical ubiquitin linkages. For example, TRIP12 and AREL1 promote the degradation of IL‐1β by assembling K27/K29/K33‐linked polyubiquitin chains,^[^ ^37^ ^]^ and ITCH has been shown to regulate substrate localization, lysosomal degradation, or activity by selectively assembling K27‐, K29‐, or K33‐linked chains.^[^ ^56^ ^]^ Through an siRNA‐based screen targeting candidate E3 ligases involved in the atypical polyubiquitylation of APC11, ITCH emerged as a strong candidate (Figure S5b, Supporting Information). Furthermore, we demonstrated that ITCH stabilizes APC11 by catalyzing K27/K29/K33‐linked polyubiquitin chain formation in a CUL5‐dependent manner. This conclusion is supported by several lines of evidence: 1) silencing ITCH reduced APC11 protein levels and shortened its half‐life; 2) ectopical expression of ITCH increased APC11 protein levels in control HeLa cells but not in CUL5‐null cells; and 3) ITCH promoted K27/K29/K33‐linked polyubiquitylation of wild‐type APC11, but not the W14A mutant that hardly binds to CUL5 (Figure 6). Together, these findings uncover a novel mechanism by which ITCH, in cooperation with CUL5, regulates APC11 stability through atypical polyubiquitin chains. Our study thus provides new mechanistic insights into the assembly and functional significance of atypical ubiquitin chains.

Similar to how APC11 depletion promotes cancer metastasis by inhibiting CRL5 activity and leading to the accumulation of integrin β1 (Figure 4; Figure S3, Supporting Information), CUL5 knockdown destabilizes APC11, thereby impairing APC/C‐associated processes. These include inhibition of mitotic exit, induction of polyploidy, and increased sensitivity of cancer cells to paclitaxel (Figure 7; Figure S6, Supporting Information). Supporting our findings, a previous study reported that CUL5 localizes to centriole—structures essential for genome stability—and that its depletion in U2OS cells led to increased polyploidy and aneuploidy.^[^ ^57^ ^]^ However, the underlying mechanism remained unclear, with the authors speculating that SRC, a known CRL5 substrate,^[^ ^58^ ^]^ might be involved. Several APC/C components and regulators are known to localize at centrosomes, where they contribute to the spatiotemporal regulation of APC/C activity.^[^ ^59^ ^]^ Given that overexpression of APC11 in our study rescued the mitotic defects caused by CUL5 knockdown, we propose that CUL5 may interact with APC11 at centrosome to regulate APC/C activity and maintain genome stability.

Notably, CRL5 has been implicated in mediating resistance to CDK9 and MCL1 inhibitors in lung cancer cells by targeting pro‐apoptotic proteins NOXA and BIM for degradation.^[^ ^60^ ^]^ Our findings extend this role by showing that CUL5 also promotes resistance to the anti‐microtubule agent paclitaxel, which stabilizes microtubules, by delaying mitotic exit. These dual functions of CUL5 appear contradictory: on one hand, it exhibits tumor‐suppressive characteristics by maintaining genome stability; on the other hand, it displays oncogenic behavior by reducing sensitivity to chemotherapeutic agents. In fact, this paradox is not unique to CUL5. For example, CUL9, which cooperates with the 3M complex to maintain genome integrity, also promotes polyploidy when deleted, yet its loss sensitizes U2OS cells to microtubule‐damaging agents such as Taxol.^[^ ^61^ ^]^ Therefore, the role of CUL5 in cancer is complex and highly context‐dependent, warranting further investigation.

In summary, our study revealed a previously uncovered interplay between two unique multi‐subunit E3 ubiquitin ligases—CRL5 and APC/C—mediated through direct interaction between CUL5 and APC11. On one hand, APC11 binds to CUL5 and inhibits its neddylation by blocking the interaction between UBE2F and CUL5. Interestingly, APC11 depletion enhances CUL5 neddylation but impairs CRL5 activity by destabilizing its receptors, such as SOCS3. As a result, integrin β1, a substrate of the CRL5^SOCS3^ E3 ligase, accumulates, promoting cancer cell migration and metastasis. Conversely, CUL5 interacts with APC11 and recruits the E3 ligase ITCH to catalyze the attachment of K27, K29, and K33‐linked polyubiquitin chains on Lys83 of APC11, thereby stabilizing APC11. Consequently, CUL5 knockdown downregulates APC11 levels, leading to reduced APC/C activity, delayed mitotic exit, and increased sensitivity to the microtubule‐targeting drug paclitaxel (Figure 7k). Together, our findings highlight the importance of maintaining appropriate levels of both CUL5 and APC11 for the proper function of CRL5 and APC/C ligases, and underscore the intricate balance between cell cycle regulation, genomic stability, and chemoresistance. Further structural characterization of APC11 in complex with CUL5 will be instrumental in fully elucidating the molecular basis by which APC11 suppresses the CUL5‐UBE2F interaction, as well as CUL5‐ITCH‐mediated K27/K29/K33‐linked polyubiquitylation. Moreover, investigating the in vivo crosstalk between CUL5 and APC11 in cancer types beyond pancreatic cancer and cervical carcinoma represents an important and intriguing direction for future research.

## 4 Experimental Section

### Cell Culture and Chemicals

HEK293, HEK293T, MIA PaCa‐2, PANC‐1, A549, Hep3B, PLC/PRF/5 and HeLa cells were cultured in Dulbecco's modified Eagle's medium (DMEM) supplemented with 10% (v/v) fetal bovine serum (FBS) and 1% penicillin/streptomycin (P/S). The primary MEF cells were isolated from day E13.5 mouse embryo and maintained in DMEM supplemented with 15% FBS, 2 mm L‐glutamine, 0.1 mm MEM nonessential amino acid and 1% P/S. HeLa cells with CUL5 knockout by CRISPR‐Cas9 were established by puromycin (Selleck, S7417) selection after transfection with a sequence‐verified CRISPR plasmid. Target sequence of sgCUL5 is 5′‐GGC GAC GTC TAA TCT GTT AA‐3′.

The chemicals were obtained from the following commercial suppliers: MLN4924 (Apexbio, B1036), CHX (Sigma–Aldrich, C7698), MG132 (Cayman, 10012628), PS341 (MCE, HY‐10227), CQ (Sigma–Aldrich, C6628), Nocodazole (Sigma–Aldrich, M1404), and Paclitaxel (MCE, HY‐B0015).

### Tandem Affinity Purification and LC‐MS/MS Analysis

MIA PaCa‐2 cells stably expressing SBP‐S‐CUL5 or the control vector pLVX were harvested for tandem affinity purification. Briefly, cells were lysed in NETN buffer (0.5% NP‐40, 50 mm Tris pH 8, 0.1 m NaCl, 2 mm EDTA) supplemented with complete protease inhibitors (Roche, 11873580001). SBP‐S‐CUL5 was first affinity‐purified using Streptavidin Sepharose (Thermo Fisher Scientific, 20359) and eluted with 2 mg/mL biotin in NETN buffer. The eluate was subsequently incubated with S‐protein agarose (Millipore, 69704) and eluted with SDS sample buffer for SDS‐PAGE and mass spectrometry analysis. The analysis was performed by Jingjie PTM BioLabs (Hangzhou, China). Gel bands (≈1 mm^3^) were decolorized, digested overnight with trypsin, reduced with DTT, alkylated with IAM, and desalted using C18 ZipTips. Peptides were separated on an EASY‐nLC 1000 system with a 0.1% FA/2% ACN (solvent A) to 0.1% FA/90% ACN (solvent B) gradient at 550 nL min^−1^ and analyzed on a Q Exactive Plus mass spectrometer (350–1800 m/z, 70000 MS1 resolution; top 20 HCD‐MS/MS, 17500 MS2 resolution, NCE 28%, 20 s dynamic exclusion). Data were searched in Proteome Discoverer v2.1 against the Homo sapiens SwissProt database (≤ 2 missed cleavages) with 10 ppm precursor and 0.02 Da fragment tolerance; Cys carbamidomethylation was set as fixed, and Met oxidation and protein N‐terminal acetylation as variable modifications. Peptides with a score >20 were considered high‐confidence identifications. The mass spectrometry proteomics data have been deposited to the ProteomeXchange Consortium via the PRIDE^[^ ^62^ ^]^ partner repository with the dataset identifier PXD068961.

### Immunoblotting (IB) and Immunoprecipitation (IP)

The cells were harvested and lysed in lysis buffer (50 mm Tris pH 7.5, 0.15 m NaCl, 1% NP‐40, 0.1% SDS, 0.5% sodium deoxycholate, 1 mm EDTA, 1 mm DTT, 50 mm NaF, 1 mm Na_3_VO_4_) with complete protease inhibitors (Roche) and phosphatase inhibitors (Roche, 04906837001). For direct IB analysis, the lysates were quantified with the BCA protein assay kit (Thermo Fisher Scientific, 23225), and equal amounts of samples were to western blotting. For exogenous IP, cell lysates were incubated with FLAG antibody‐conjugated beads (Sigma–Aldrich, A2220) for 3 h. For endogenous IP, cell lysates were incubated with anti‐CUL5 antibody (Santa Cruz, sc‐373822) or normal mouse IgG (Santa Cruz, sc‐2025) for CUL5 IP, and with anti‐ITCH antibody (Proteintech, 20920‐1‐AP) or normal rabbit IgG (Santa Cruz, sc‐2027) for ITCH IP, each for 3 h, followed by additional 3 h incubation with protein G sepharose (Cytiva, 17061801). The immunoprecipitates were washed four times with lysis buffer and then subjected to western blotting.^[^ ^63^ ^]^

The following antibodies were used: ACTIN (Sigma–Aldrich, A5441), FLAG (Sigma–Aldrich, F1804), FLAG (Sigma–Aldrich, F7425), GFP (Santa Cruz, sc‐9996), MYC (Santa Cruz, sc‐789), APC2 (Cell Signaling Technology, 12301), APC8 (Cell Signaling Technology, 15100), APC10 (Cell Signaling Technology, 14807), APC11 (Cell Signaling Technology, 14090), APC11 (Santa Cruz, sc‐517142), SAG (Proteintech, 11905‐1‐AP), RBX1 (Proteintech, 14895‐1‐AP), CUL1 (Santa Cruz, sc‐11384), CUL5 (Abcam, ab184177), CUL5 (Santa Cruz, sc‐373822), CAND1 (Abcam, ab183748), integrin β1 (Cell Signaling Technology, 34 971), p‐SRC (Y416) (Cell Signaling Technology, 6943), ATM (Cell Signaling Technology, 2873), Cyclin B1 (Cell Signaling Technology, 4138), RPB1 (Cell Signaling Technology, 14958), EGFR (Cell Signaling Technology, 4267), DEPTOR (Cell Signaling Technology, 11 816), NOXA (Millipore, OP180), UBE2F (Proteintech, 17056‐1‐AP), COPS5 (Cell Signaling Technology, 6895), SOCS3 (Proteintech, 14025‐1‐AP), LC3 (Cell Signaling Technology, 2775), securin (Cell Signaling Technology, 13445), ITCH (Proteintech, 20920‐1‐AP), Cyclin E1 (Cell Signaling Technology, 20808), p‐H3 (Cell Signaling Technology, 3377), and p‐Aurora A/B/C (Cell Signaling Technology, 2914), Aurora A (Cell Signaling Technology, 14 475), Aurora B (Abcam, ab2254), and NEDD8 (Abcam, ab81264).

### siRNA and Lentiviral shRNA Silencing

Cells were transfected with the following siRNA oligos by Lipofectamine 2000 (Invitrogen, 11668019) to achieve transient knockdown of endogenous genes. siCtrl: 5′‐ATT GTA TGC GAT CGC AGA C‐3′; siCUL5: 5′‐GCT AGA ATG TTT CAG GAC ATA‐3′; siCUL5‐2: 5′‐GTC TCA CTT CCT ACT GAA CTG‐3′; siAPC11: 5′‐TCT GCA GGA TGG CAT TTA A‐3′; siAPC11‐2: 5′‐CCA CAT GCA TTG CAT CCT CAA‐3′; siAPC11‐3: 5′‐ACT CAT TAA ACT ACT CAA ATC‐3′; siAPC2: 5′‐TGC GCG GAG TCT TGT TCT TTA‐3′; siAPC2‐2: 5′‐GCA GAT TAA AGC AAG TCA GAT‐3′; siSAG: 5′‐CCT GTG GGT GAA ACA GAA CAA‐3′; siSAG‐2: 5′‐CGA CAA GAT GTT CTC CCT CAA‐3′; siUBE2F: 5′‐CAA AGT GAA ATG CCT GAC CAA‐3′; siUBE2F‐2: 5′‐CAT CAA ACG TTA TGC CAG AT‐3′; siintegrin β1: 5′‐GCC CTC CAG ATG ACA TAG AAA‐3′; siintegrin β1‐2: 5′‐GCC TTG CAT TAC TGC TGA TAT‐3′; siITCH: 5′‐GCC TAT GTT CGG GAC TTC AAA‐3′; siITCH‐2: 5′‐GGT GAC AAA GAG CCA ACA GAG‐3′; siWSB1: 5′‐AGT TTC TCT CGT ATC GTA TTT‐3′; siAREL1: 5′‐CCG GGA ATG GTT TGA GCT AAT‐3′; siTRIP12: 5′‐TCG CAA AGG TTA AGA TGA A‐3′; siTRAF4: 5′‐CCA GGA CAT TCG AAA GCG AAA‐3′; siCOPS5: 5′‐GCT CAG AGT ATC GAT GAA A‐3′; and siCOPS5‐2: 5′‐CAG TCT CTG AGA AGT ACT TTA‐3′. Short hairpins targeting APC11 (targeting sequence: 5′‐TCT GCA GGA TGG CAT TTA A‐3′), CUL5 (targeting sequence: 5′‐GCT AGA ATG TTT CAG GAC ATA‐3′) and integrin β1 (targeting sequence: 5′‐GCC CTC CAG ATG ACA TAG AAA‐3′) were cloned into pLKO.1‐puro vector and packaged with pMD2.G and pspAX2 plasmids to generate lentiviral shRNA viruses. Cells were infected with the indicated lentiviruses and then selected with puromycin for stable knockdown.

### Quantitative Real‐Time Reverse‐Transcription PCR

Total RNA was isolated from cells using TRIzol reagent (Invitrogen, 15596018) and then transcribed into complementary DNA using the PrimeScript RT reagent kit (Takara, RR037A). Quantitative real‐time reverse‐transcription PCR (qRT‐PCR) was performed with SYBR Premix Ex Taq (Takara, RR420A) on the CFX96 Real‐time PCR System (Bio‐Rad). Relative mRNA levels of *integrin β1* or *APC11* were determined by normalization to the housekeeping gene *GAPDH* using the comparative Ct (2^−ΔΔCt^) method. The primers used for qRT‐PCR were as follows: 5′‐AAC TAC GAA TAC TAA GAA CCC AGG AA‐3′ and 5′‐CAC TCT ATT TGC TCT TTT ATC ATT TTC T‐3′ for *CUL5*; 5′‐GCC AAA TGG GAC ACG GGT‐3′ and 5′‐CAA GAG TAA CCA TCC TGT CTC AAG TC‐3′ for *integrin β1*; 5′‐ATG CTG CCC TGA CTG CAA G‐3′ and 5′‐ATG GCA AAA CAA ACG CAG C‐3′ for *APC11*; 5′‐AGG GCA TCC TGG GCT ACA C‐3′ and 5′‐GCC AAA TTC GTT GTC ATA CCA G‐3′ for *GAPDH*.

### The In Vitro Binding Assay

Recombinant HIS‐APC11 protein was purified from *E.coli* BL21(DE3) (TransGen, CD701) with HIS‐tag Protein Purification Kit (Beyotime, P2226) according to the manufacturer's instructions. For the in vitro binding assay, HEK293 cells transfected with a mock vector or FLAG‐CUL5 plasmid were lysed and incubated with FLAG antibody‐conjugated beads for 3 h. The beads were washed four times with lysis buffer and then incubated with purified HIS‐APC11 protein for another 3 h. Finally, the beads were washed four times with lysis buffer, boiled, and subjected to IB with indicated Abs.^[^ ^64^ ^]^

### The In Vivo Ubiquitylation Assay

HEK293 cells were transfected with the indicated plasmids with PolyJet (SignaGen Laboratories, SL100688) for 48 h, and then harvested for the in vivo ubiquitylation assay. The cells were lysed in the guanidine denaturing solution (6 m guanidinium‐HCl, 0.1 m Na_2_HPO4, 5 mm imidazole, 10 mm β‐mercaptoethanol, 10 mm Tris pH 8.0) and sonicated. Subsequently, the lysates were incubated with nickel‐nitrilotriacetic acid (Ni‐NTA) agarose (QIAGEN, 1018244) for HIS‐tagged protein purification, as described previously.^[^ ^65^ ^]^

### Transwell Migration Assay

PLC/PRF/5 cells (1 × 10^5^) or A549 cells (1 × 10^4^) in 200 µL serum free medium were seeded in an 8 µm pores, 24‐well plate chamber insert (Corning, 3422) coated with Matrigel (Corning, 356234), while medium with 10% FBS was supplemented at the bottom of the insert. After incubating for 12 h (A549 cells) or 24 h (PLC/PRF/5 cells), cells were fixed with 4% paraformaldehyde for 10 min, and stained with 0.1% crystal violet for 20 min. Cells on the upper surface of the insert were removed with a cotton swab. The positively stained cells on the underside of the filters were photographed using the microscope (OLYMPUS, IX73). The number of migratory cells in three random fields per insert was counted.

### Cell Viability Assay

HeLa cells infected with indicated lentiviruses or transfected with indicated siRNA oligos were seeded in 96‐well plates at 2 × 10^3^ cells per well in triplicate. Cells were treated with paclitaxel at various concentrations for 72 h, and then subjected to the Cell Counting Kit 8 (CCK8) assay according to the manufacturer's instructions (MCE, HY‐K0301).

### FACS Analysis

HeLa cells after various treatments were harvested by trypsinization and fixed in ice‐cold 70% ethanol overnight. Cells were then stained with propidium iodide (PI) buffer (BD, 550825) and analyzed by flow cytometry (Beckman, CytoFLEX S).

### Immunofluorescent Staining

The indicated cells were fixed with 4% paraformaldehyde for 10 min, subsequently permeabilized with 0.5% Triton X‐100 for 10 min, and subjected to incubation with a blocking buffer (PBS containing 2.5% FBS, 0.5% BSA, and 0.05% Triton X‐100) for 30 min. Afterward, cells were immunostained with an α‐tubulin antibody (Sigma–Aldrich, T8203) for 1 h, followed by staining with secondary antibody conjugated with Alexa Fluor 488 (Invitrogen, A‐11001) for 1 h, and DAPI (Beyotime, C1002) for an additional 30 min at room temperature. Images were captured on the confocal fluorescence microscope (Nikon, Nikon A1 Ti).^[^ ^66^ ^]^

### Animal Experiments

All mice procedures were approved by the Animal Care and Use Committee of Zhejiang University. For the in vivo metastasis assay, PLC/PRF/5 cells (4 × 10^6^) stably expressing the indicated shRNA were injected into the tail vein of 5‐week‐old female BALB/c nude mice (GemPharmatech). Six weeks after injection, the mice were sacrificed, and all the lung tissues were collected for hematoxylin and eosin (H&E) staining. Macroscopic metastases were quantified by counting metastasis tumors in all five lung lobes from each mouse.

To evaluate the therapeutic effect of paclitaxel in vivo, HeLa cells (1 × 10^6^) stably expressing indicated shRNA and plasmid were suspended in 100 µL PBS and then injected subcutaneously into the flank of 5‐week‐old female BALB/c nude mice. When the tumor reached the volume of ≈50 mm^3^, the mice were randomly divided into two groups and administered intraperitoneal injection of 15 mg/kg paclitaxel or an equivalent volume of vehicle (Cremophor EL/ethanol 1:1, diluted 1:4 with PBS) three times a week. The body weight, tumor length (*L*), and the width (*W*) were measured each time prior to the injection. After 18 days, tumors were collected, weighed, and photographed. Tumor volumes (*V*) were calculated with the formula *V* = (*L* × *W^2^*) × 0.5.

### IHC Staining

The 5‐µm‐thick sections of mouse pancreatic tissues were stained with H&E, anti‐APC11 antibody (Cell Signaling Technology, 14090), or anti‐integrin β1 antibody (Cell Signaling Technology, 34971), as previously described.^[^ ^67^ ^]^ The human pancreatic cancer tissue microarrays were obtained from Taibsbio (Xi'an, China) and immunostained with anti‐APC11 antibody or anti‐integrin β1 antibody. Slides were scanned using a section scanner (KFBIO, KF‐FL‐020).

### Statistical Analysis

The data were collected from three independent biological experiments and presented as the mean ± SEM. The significance of cell viability assays in vitro and in vivo was determined by the two‐way repeated‐measures ANOVA analysis using the GraphPad Prism software (Version 8.3, San Diego, CA, USA). Differences in APC11 and integrin β1 expression between metastatic and non‐metastatic human pancreatic cancer tissues were analyzed using the Wilcoxon rank–sum test. Other statistical analyses were determined by Student's *t*‐test using the Statistical Program for Social Sciences software (SPSS 20.0, Chicago, IL, USA). *p* < 0.05 was considered statistically significant.

## Conflict of Interest

The authors declare no conflict of interest.

## Author Contributions

D.C., R.Q., and T.L. contributed equally to this work and also co‐first authors. D.C. designed and performed the experiments, analyzed and interpreted the data, and drafted the manuscript. R.Q., T.L., L.W., X.C., S.S., and X.L. performed the experiments. J.X. analyzed and interpreted the data. Y.S. revised the manuscript. X.X. analyzed and interpreted the data and revised the manuscript. Y.Z. designed the study, analyzed and interpreted the data, and revised and finalized the manuscript. All authors reviewed the manuscript.

## Supporting information



Supporting Information

## Data Availability

The data that support the findings of this study are available from the corresponding author upon reasonable request.
