# Supplementary material for: The Crosstalk Between CRL5 and APC/C E3 Ligases Regulates Metastasis and Chemosensitivity of Cancer Cells
Source: Adv Sci (Weinh). 2025 Oct 29;13(3):e12652. doi: 10.1002/advs.202512652 (PMC12806337; doi:10.1002/advs.202512652)

Supplemental information

Supplementary figure legends

Supplementary Fig.1 APC11 does not compete with SAG for CUL5 binding. A549 and PLC/PRF/5 cells were transfected with distinct siRNA oligos targeting APC11, SAG or scrambled control siRNA for 72 h, followed by immunoprecipitation using either IgG or anti-CUL5 Ab.

Supplementary Fig. 2 APC11 depletion promotes CUL5 neddylation but stabilizes integrin β1. a Immunoblot of indicated proteins in A549 cells upon APC11 knockdown with siRNA oligos. **b** qRT-PCR analysis of CUL5 mRNA levels in Hep3B and PLC/PRF/5 cells following APC11 knockdown. Data are presented as mean ± SEM, n = 3; ns, not significant.. **c** Immunoblot of proteins associated with CUL5 neddylation in A549 and Hep3B cells following APC11 knockdown with two distinct siRNA oligos. **d** Co-IP of ectopically expressed FLAG-CUL5 and endogenous UBE2F, CAND1, COPS5, or SAG in HEK293 cells following transfection with indicated distinct siRNA oligos. **e** Co-IP of endogenous CUL5 with UBE2F or APC11 in A549 cells transfected with another specific siRNA oligo targeting APC11. **f** qRT-PCR analysis to assess mRNA levels of integrin β1 in APC11 knockdown Hep3B and PLC/PRF/5 cells. mean ± SEM, n = 3, ns, not significant. **g**, **h** Degradation of SOCS3 and integrin β1 in APC11-depleted cells. A549 and MIA PaCa-2 cells were transfected with an additional APC11-specific siRNA oligo (**g**), while PANC-1 cells were transfected with two distinct APC11-targeting siRNA oligos (**h**). Cells were then treated with CHX (100 μg/mL) for the indicated time periods, followed by IB analysis. **i** Immunoblot of indicated proteins in A549 and PANC-1 cells upon COPS5 knockdown with two distinct siRNA oligos.

Supplementary Fig. 3 APC11 depletion facilitates cell migration by suppressing integrin β1 degradation, independently of the APC/C complex. PLC/PRF/5 (**a**, **c**, **d**) or A549 (**b**) cells were transfected with indicated siRNA oligos or plasmids for 48 h, followed by transwell cell migration assay. The siRNA oligo siAPC11-3 was designed to target the 3′-UTR of APC11, thereby avoiding suppression of FLAG-APC11 expression (**d**). Representative migration images from one out of three

biological replicates are shown **a** and **c**, left; **b** and **d**, top). Scale bar, 100 μm. The number of migration cells was counted in three random fields per chamber (**a** and **c**, right; **b** and **d**, bottom). Data are shown as mean ± SEM, n = 3, *** $p < 0.001$, ns, not significant. **e** Pancreata were harvested from *Kras*^{G12D}*;Pdx1-cre+* male mice, with or without liver metastases, at approximately 15 months of age, followed by H&E staining and immunohistochemistry using anti-APC11 and anti-integrin β1 Abs. Representative images are shown (scale bar, 60 μm) (**e**, left), and the staining quantification was analyzed by IHC scoring using an IRS system from five random fields and presented as mean ± SD (**e**, right). **f** The correlation between APC11 mRNA levels and survival probability in PAAD (pancreatic adenocarcinoma) patients from the TCGA database (https://www.proteinatlas.org/), shown as the Kaplan–Meier Plotter curve (low levels, n = 45; high levels, n = 131).

Supplementary Fig. 4 CUL5 stabilizes APC11 protein levels. a, **b** Immunoblot of APC11 and other APC/C complex components in Hep3B cells transfected with wild-type CUL5 for 48 h (**a**), and in HEK293 cells transfected with the NEDD8 modification-deficient CUL5 mutant (K724R) for 48 h (**b**). **c**, **d** Hep3B and PLC/PRF/5 cells were transfected with indicated siRNA oligos for 72 h (**c**) or HEK293 cells were transfected with indicated amount of FLAG-CUL5 plasmid for 48 h (**d**), followed by qRT-PCR analysis. Data are shown as mean ± SEM, n = 3, * $p < 0.05$, ** $p < 0.01$, ns, not significant. **e** The stability of APC11 in HEK293 cells upon CUL5 knockdown. HEK293 cells were transfected with indicated siRNA oligos and then treated with CHX (100 μg/mL) for the indicated time periods, followed by IB analysis. LEX: longer exposure.

Supplementary Fig. 5 ITCH ubiquitylates APC11 at lysine 83 (K83). a Immunoblot of APC11 in Hep3B and PLC/PRF/5 cells following treatment with various concentrations of MLN4924 for 24 h. **b** Immunoblot of APC11 in Hep3B cells following transfection with siRNA oligos targeting indicated E3 ligases. **c** Degradation of APC11 in ITCH-knockdown cells. Hep3B and PLC/PRF/5 cells were transfected with an additional ITCH-specific siRNA oligo for 72 h, then treated with CHX (100 μg/mL) for the indicated time periods, followed by IB analysis. **d**, **e**

Immunoblot of various FLAG-tagged APC11 K→R mutants in HEK293 cells with (**e**) or without (**d**) 6 h treatment of 20 μM MG132.

Supplementary Fig. 6 CUL5 disruption impairs mitotic exit and sensitizes cells to paclitaxel. a–c HeLa (**a**, **b**) or Hep3B (**c**) cells were transfected with indicated siRNA oligos for 48 h, and then synchronized in M phase by 100 ng/mL nocodazole treatment for 18 h. Next, the cells released into the cell cycle for different time periods, followed by IB with indicated Abs (**a**, **c**) or FACS analysis (**b**). **d** The CCK8 assays were employed to assess the sensitivity of HeLa cells transfected with indicated siRNA oligos to paclitaxel treatment. Data are presented as mean ± SEM from three independent experiments. *** $p < 0.001$.

Cui et al Figure S1

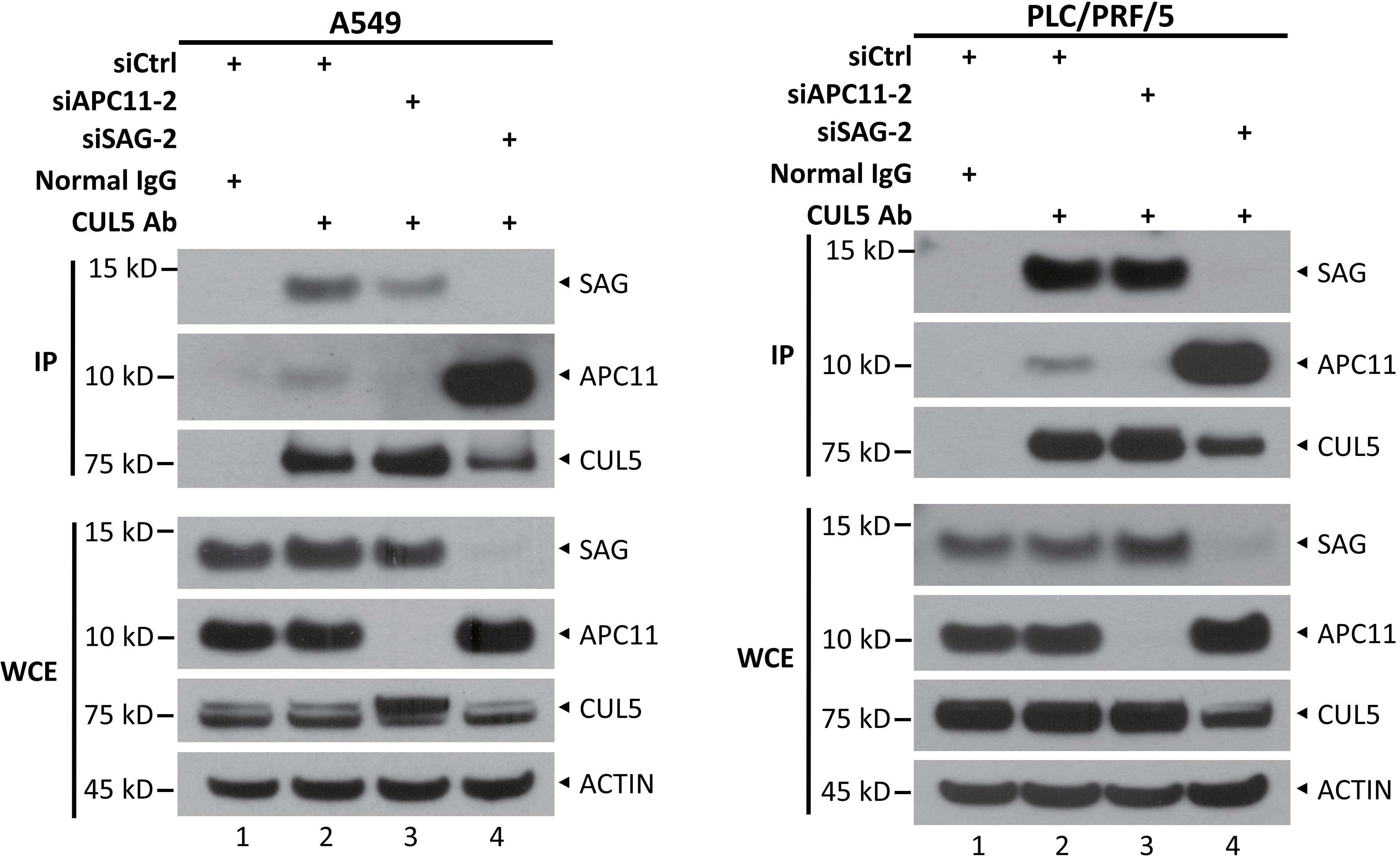

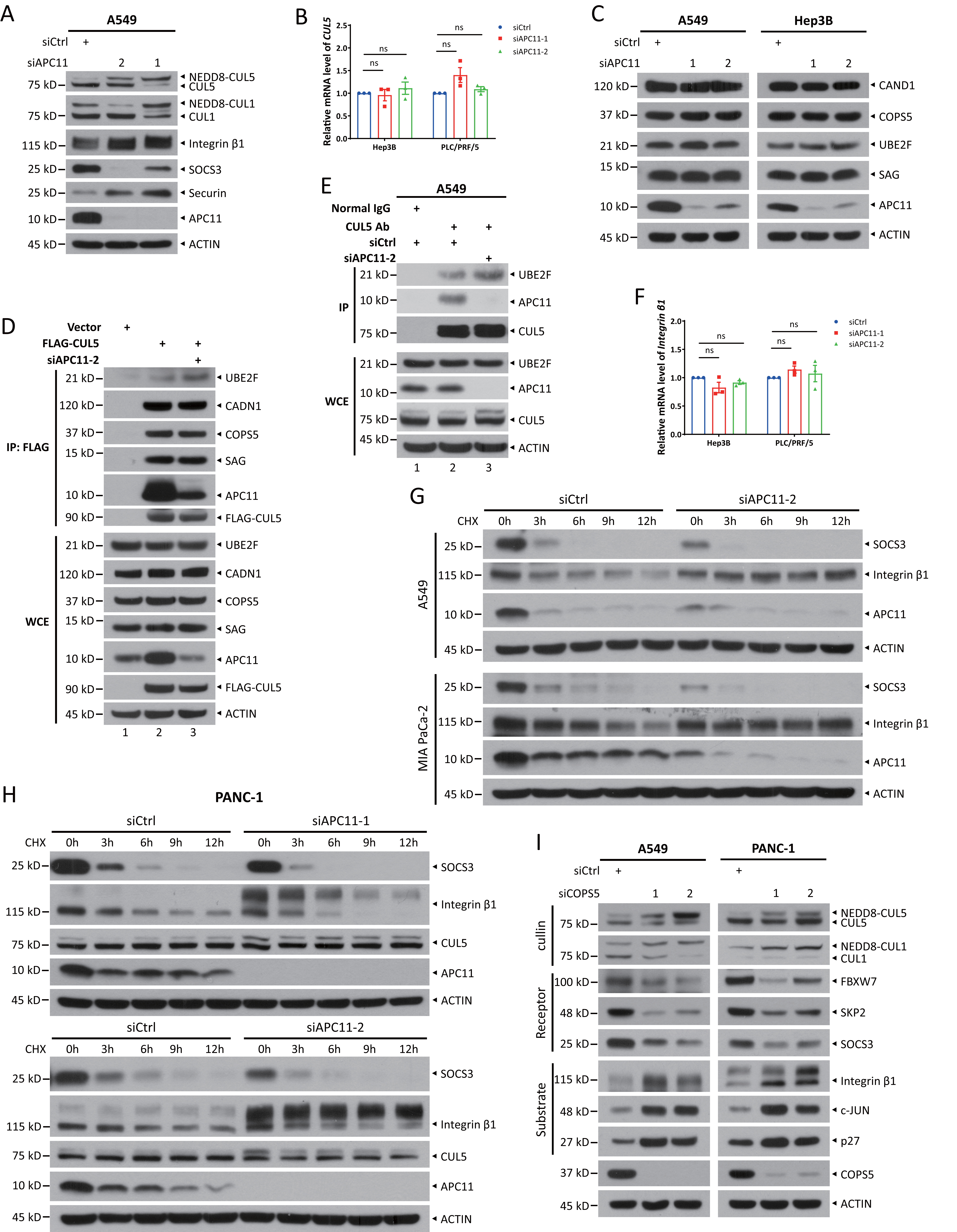
A
A549
siCtrl +
siAPC11 2 1
75 kD NEDD8-CUL5 CUL5
75 kD NEDD8-CUL1 CUL1
115 kD Integrin β1
25 kD SOCS3
25 kD Securin
10 kD APC11
45 kD ACTIN
B
Relative mRNA level of CUL5
2.5 2.0 1.5 1.0 0.5 0.0
ns ns ns ns
Hep3B PLC/PRF/5
siCtrl siAPC11-1 siAPC11-2
C
A549 Hep3B
siCtrl + +
siAPC11 1 2 1 2
120 kD CAND1
37 kD COPS5
21 kD UBE2F
15 kD SAG
10 kD APC11
45 kD ACTIN
D
Vector +
FLAG-CUL5 + +
siAPC11-2 +
IP: FLAG
21 kD UBE2F
120 kD CADN1
37 kD COPS5
15 kD SAG
10 kD APC11
90 kD FLAG-CUL5
WCE
21 kD UBE2F
120 kD CADN1
37 kD COPS5
15 kD SAG
10 kD APC11
90 kD FLAG-CUL5
45 kD ACTIN
1 2 3
E
A549
Normal IgG +
CUL5 Ab + +
siCtrl + +
siAPC11-2 +
IP
21 kD UBE2F
10 kD APC11
75 kD CUL5
WCE
21 kD UBE2F
10 kD APC11
75 kD CUL5
45 kD ACTIN
1 2 3
F
Relative mRNA level of Integrin β1
2.0 1.5 1.0 0.5 0.0
ns ns ns ns
Hep3B PLC/PRF/5
siCtrl siAPC11-1 siAPC11-2
G
siCtrl siAPC11-2
CHX 0h 3h 6h 9h 12h 0h 3h 6h 9h 12h
A549
25 kD SOCS3
115 kD Integrin β1
10 kD APC11
45 kD ACTIN
MIA PaCa-2
25 kD SOCS3
115 kD Integrin β1
10 kD APC11
45 kD ACTIN
H
PANC-1
siCtrl siAPC11-1
CHX 0h 3h 6h 9h 12h 0h 3h 6h 9h 12h
25 kD SOCS3
115 kD Integrin β1
75 kD CUL5
10 kD APC11
45 kD ACTIN
siCtrl siAPC11-2
CHX 0h 3h 6h 9h 12h 0h 3h 6h 9h 12h
25 kD SOCS3
115 kD Integrin β1
75 kD CUL5
10 kD APC11
45 kD ACTIN
I
A549 PANC-1
siCtrl + +
siCOPS5 1 2 1 2
cullin
75 kD NEDD8-CUL5 CUL5
75 kD NEDD8-CUL1 CUL1
Receptor
100 kD FBXW7
48 kD SKP2
25 kD SOCS3
Substrate
115 kD Integrin β1
48 kD c-JUN
27 kD p27
37 kD COPS5
45 kD ACTIN

Cui et al Figure S3

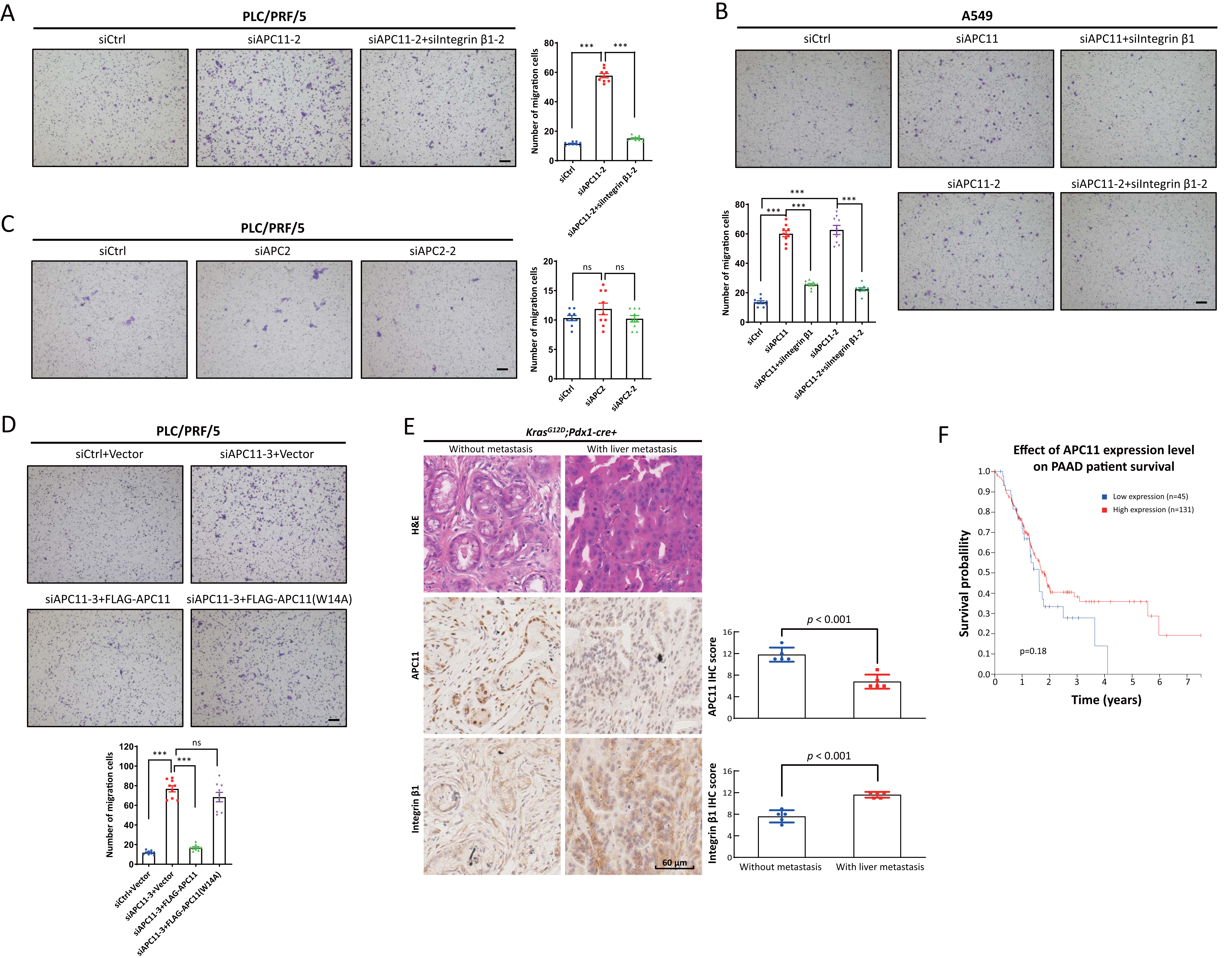

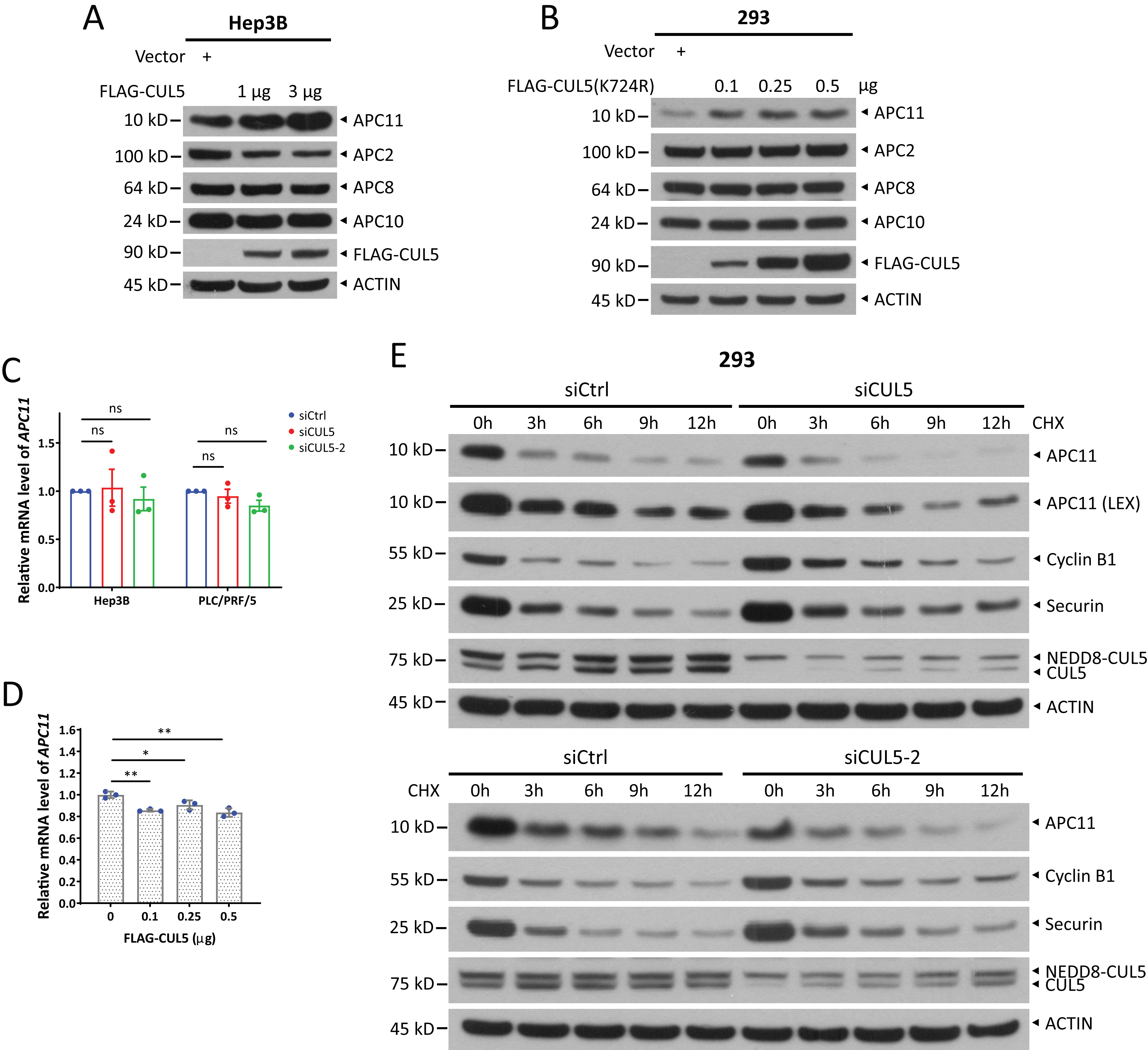
A
Hep3B
Vector +
FLAG-CUL5 1 µg 3 µg
10 kD APC11
100 kD APC2
64 kD APC8
24 kD APC10
90 kD FLAG-CUL5
45 kD ACTIN
B
293
Vector +
FLAG-CUL5(K724R) 0.1 0.25 0.5 µg
10 kD APC11
100 kD APC2
64 kD APC8
24 kD APC10
90 kD FLAG-CUL5
45 kD ACTIN
C
Relative mRNA level of APC11
siCtrl
siCUL5
siCUL5-2
ns
Hep3B
PLC/PRF/5
D
Relative mRNA level of APC11
**
*
FLAG-CUL5 (µg)
0 0.1 0.25 0.5
E
293
siCtrl
siCUL5
0h 3h 6h 9h 12h CHX
10 kD APC11
10 kD APC11 (LEX)
55 kD Cyclin B1
25 kD Securin
75 kD NEDD8-CUL5
CUL5
45 kD ACTIN
siCUL5-2
CHX
APC11
Cyclin B1
Securin
NEDD8-CUL5
CUL5
ACTIN

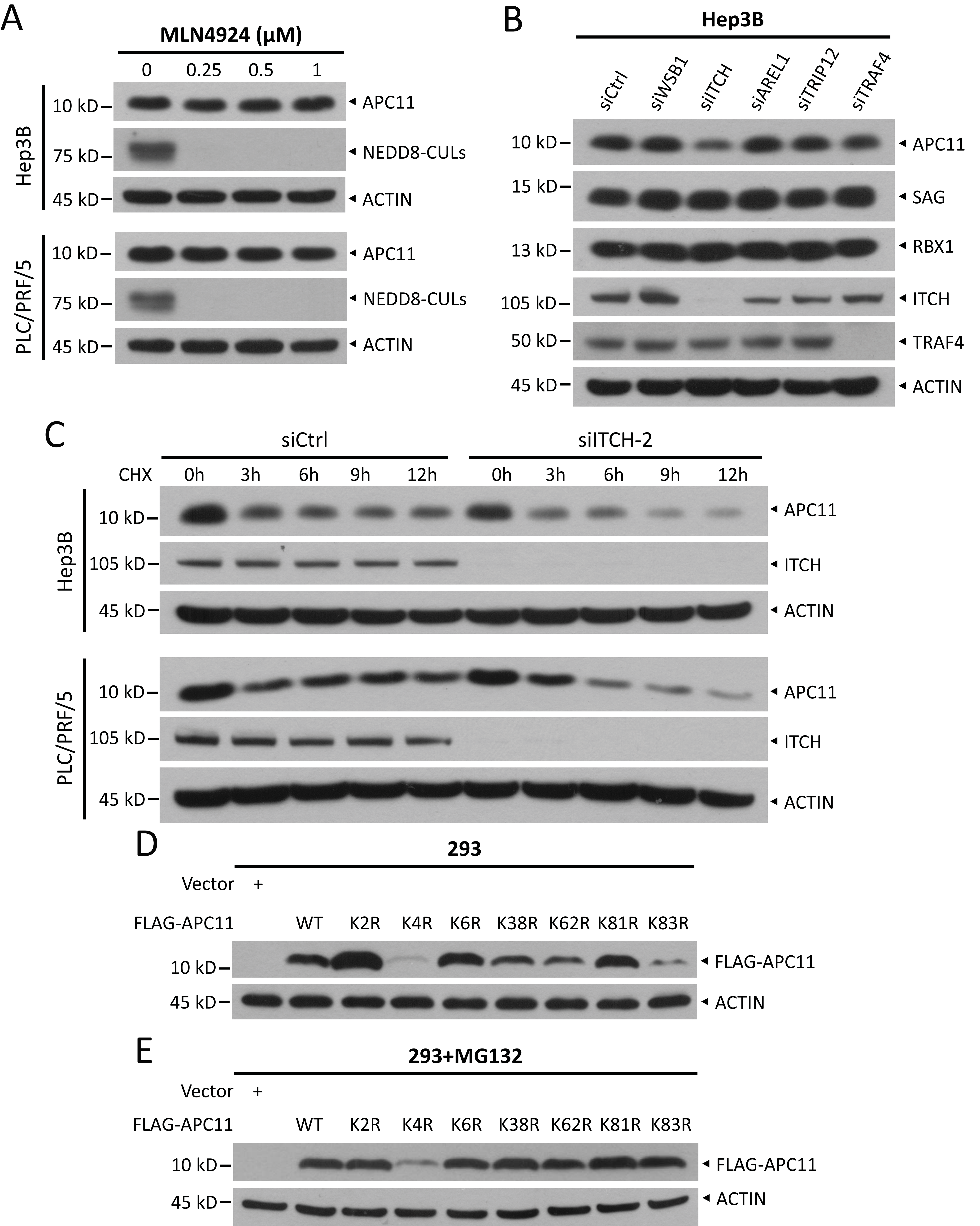
A
MLN4924 (μM)
0
0.25
0.5
1
Hep3B
10 kD
75 kD
45 kD
APC11
NEDD8-CULs
ACTIN
PLC/PRF/5
10 kD
75 kD
45 kD
APC11
NEDD8-CULs
ACTIN
B
Hep3B
siCtrl
siWSB1
siITCH
siAREL1
siTRIP12
siTRAF4
10 kD
15 kD
13 kD
105 kD
50 kD
45 kD
APC11
SAG
RBX1
ITCH
TRAF4
ACTIN
C
siCtrl
siITCH-2
CHX
0h
3h
6h
9h
12h
Hep3B
PLC/PRF/5
10 kD
105 kD
45 kD
APC11
ITCH
ACTIN
D
293
Vector
+
FLAG-APC11
WT
K2R
K4R
K6R
K38R
K62R
K81R
K83R
10 kD
45 kD
FLAG-APC11
ACTIN
E
293+MG132

Cui et al Figure S6

A

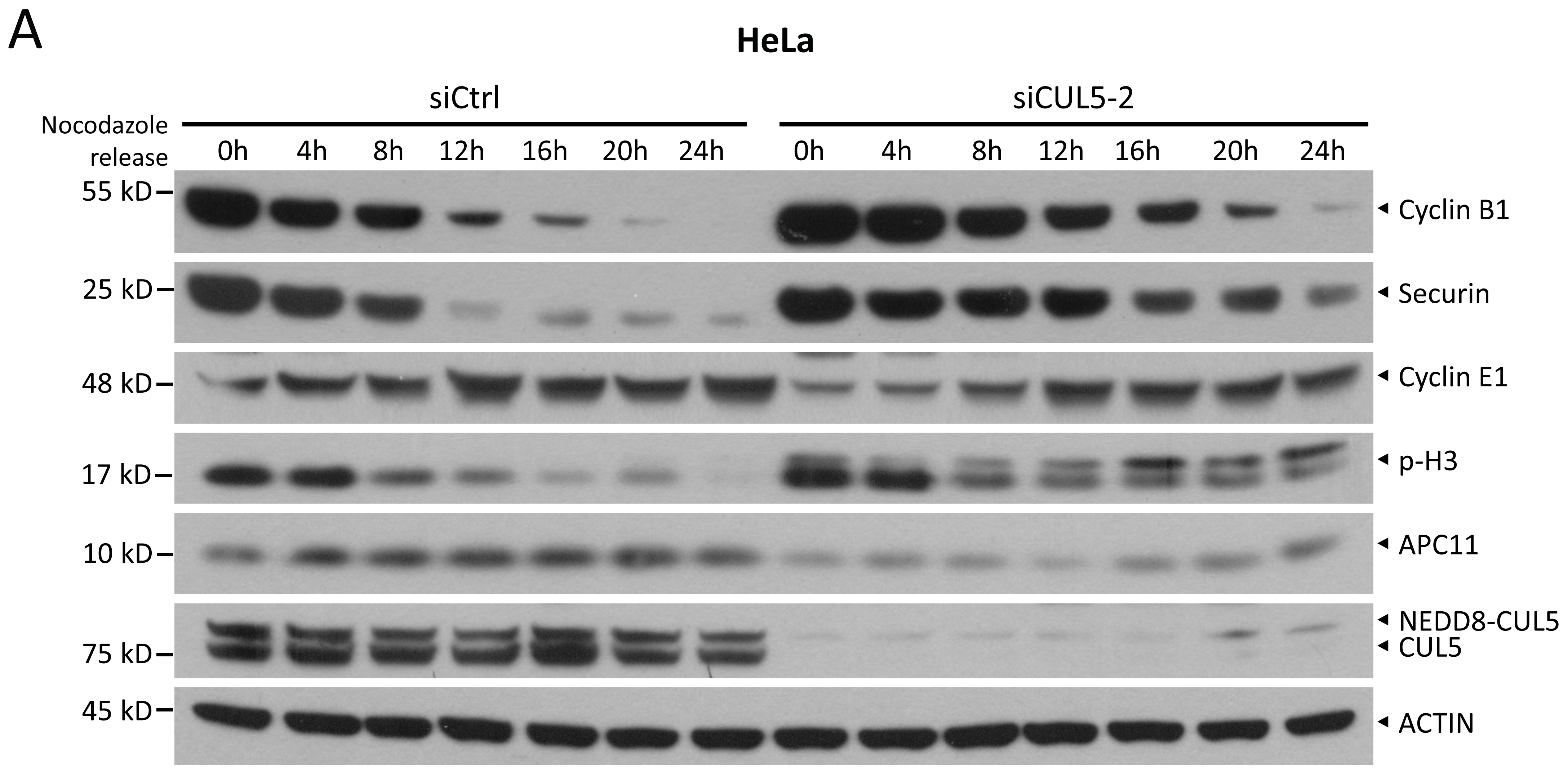

B

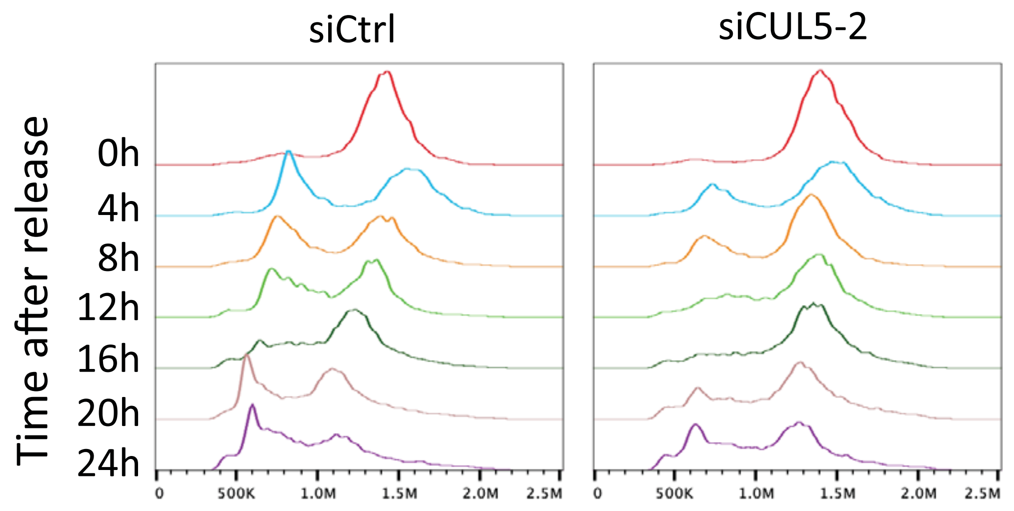

After release (h)		0	4	8	12	16	20	24
siCtrl	G2/M(%)	81.31	46.51	39.99	30.29	49.71	50.5	45.25
siCUL5	G2/M(%)	83.62	63.28	60.35	62.38	63.34	57.52	51.36

C

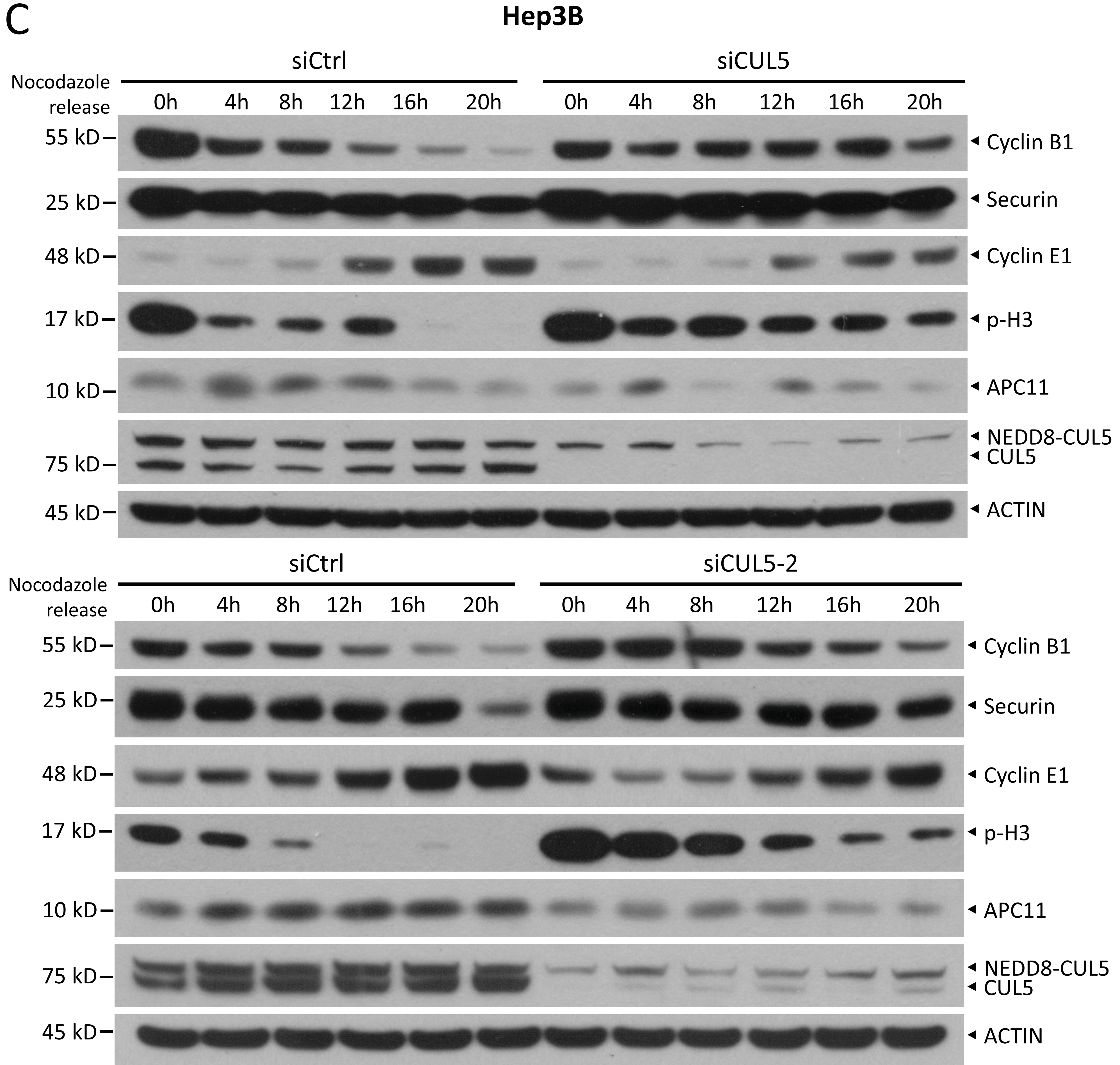

D

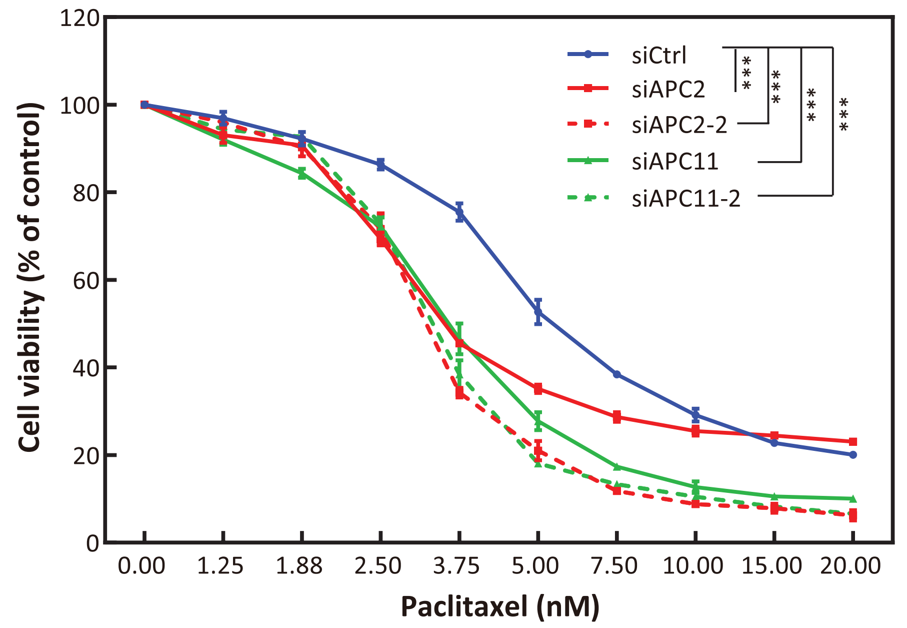

Supplement: Supplementary file 1 — Supporting Information [file ADVS-13-e12652-s001.pdf]
